# AAV mini-dystrophin gene therapy for Duchenne muscular dystrophy: a phase 1b trial

**DOI:** 10.1038/s41591-025-03750-3

**Published:** 2025-06-27

**Authors:** Russell J. Butterfield, Perry B. Shieh, Huihua Li, Michael Binks, Tara G. McDonnell, Kelly A. Ryan, Marielle Delnomdedieu, Beth A. Belluscio, Srividya Neelakantan, Daniel I. Levy, Pamela F. Schwartz, Edward C. Smith

**Affiliations:** 1 University of Utah School of Medicine, Salt Lake City, UT USA; 2 University of California at Los Angeles (UCLA), Los Angeles, CA USA; 3 Pfizer Inc, New York, NY USA; 4 Duke University School of Medicine, Durham, NC USA

**Keywords:** Neuroscience, Molecular neuroscience, Diseases of the nervous system

## Abstract

Gene therapy represents a promising approach for Duchenne muscular dystrophy (DMD), a rare X-linked genetic muscle disease. Fordadistrogene movaparvovec (PF-06939926) is an adeno-associated virus serotype 9 gene therapy containing a miniaturized dystrophin being developed for DMD, which aims to restore functional protein to muscle. We present 1-year data from ambulatory and nonambulatory participants in a phase 1b, multicenter, single-arm, open-label trial. Pediatric ambulatory male participants with a genetic DMD diagnosis and receiving stable glucocorticoids received a single intravenous low-dose (*n* = 3) or high-dose (*n* = 16) fordadistrogene movaparvovec. The primary endpoint was safety and tolerability at 1 year after dosing. In the ambulatory group, mean ± s.d. age at dosing was 8.6 ± 1.6 years. The most common treatment-emergent adverse events in the ambulatory group were vomiting (*n* = 15), nausea (*n* = 10), thrombocytopenia (*n* = 9), pyrexia (*n* = 9), decreased appetite (*n* = 8), fatigue (*n* = 7) and headache (*n* = 7). Three treatment-related serious adverse events occurred after dosing (dehydration, acute kidney injury, thrombocytopenia; all resolved within 15 days). In a small nonambulatory group (*n* = 3), mean ± s.d. age at dosing was 15.1 ± 1.0 years. The most common treatment-emergent adverse events were nausea (*n* = 3), vomiting (*n* = 3) and headache (*n* = 3); two severe treatment-related adverse events (hemolytic uremic syndrome and fatal cardiogenic shock) were observed. In the high-dose ambulatory group, the secondary endpoint of mini-dystrophin quantification showed robust expression. Mean (95% confidence interval) percent of mini-dystrophin-positive fibers for baseline, 2 months and 1 year were 0.1% (0.1–0.2), 20.3% (12.2–29.3) and 34.8% (21.1–49.8), respectively. At the 1-year time point of primary completion, fordadistrogene movaparvovec demonstrated an acceptable safety profile in the ambulatory population. Larger trials are needed to assess the efficacy of the gene therapy in DMD. ClinicalTrials.gov registration no. NCT03362502.

## Abstract (web summary)

In the primary report of a phase 1b trial, adeno-associated virus gene therapy with fordadistrogene movaparvovec in patients with Duchenne muscular dystrophy was well tolerated in an ambulatory cohort, and mini-dystrophin expression was increased in the high-dose cohort.

## Main

Duchenne muscular dystrophy (DMD) is an X-linked recessive disorder caused by mutations in the *DMD* gene encoding dystrophin, a protein that protects muscle from damage during contraction^1,2^. The estimated prevalence of DMD is up to 20 per 100,000 live male births^3^. Functional dystrophin is absent or very low in the muscle of individuals with DMD^1,4^. Progressive weakness begins in early childhood, loss of ambulation typically occurs by 12 years of age, and respiratory or cardiac failure leads to premature death between 20 and 40 years of age, typically from cardiac or respiratory complications^5^.

Gene therapies aim to restore functional dystrophin to skeletal and cardiac muscle as the most direct means of addressing the underlying pathology and, thereby, slowing or preventing muscle degeneration. Adeno-associated virus (AAV) vectors, which are under investigation as a gene-based therapy for a variety of conditions^6^, are based on a nonpathogenic, nonintegrative, replication-deficient member of the parvovirus family and are attractive candidates for gene therapy because of their transduction efficiency, long-term transgene expression and favorable safety and immunogenicity profile^7^. Gene therapy delivering full-length dystrophin is not possible with an AAV vector because the capsid size restricts the transgene length to ~4,000 base pairs. Miniaturized dystrophins have been developed to contain critically important domains that allow for the restoration of normal function in animal models^8–10^. Clinical studies are evaluating gene therapies as potential treatment options for DMD, with delandistrogene moxeparvovec recently granted accelerated approval by the U.S. Food and Drug Administration for use in ambulatory patients aged 4–5 years with a confirmed mutation in the *DMD* gene^11^.

Fordadistrogene movaparvovec (PF-06939926, Pfizer) is a recombinant AAV serotype 9 (rAAV9) vector with a transgene containing a codon-optimized construct of essential domains of the human *DMD* gene and a muscle-specific synthetic promoter (hybrid creatine kinase) as per Supplementary Fig. 1. Preclinical studies have shown that this vector produces a miniaturized but functional form of dystrophin (hereafter referred to as ‘mini-dystrophin’)^12,13^, which is expressed in cardiac and skeletal muscle. For clarity, the mini-dystrophin protein expressed by the fordadistrogene movaparvovec transgene contains some differences in dystrophin domains from other ‘micro-dystrophins’ in clinical development, but is approximately the same size as the others.

This first-in-human trial evaluated the safety and tolerability of a single intravenous infusion of fordadistrogene movaparvovec in participants with DMD. Other objectives included assessment of mini-dystrophin expression and distribution in muscle fibers and assessments of motor function. We present the analysis of the primary safety endpoint in all participants and other secondary and exploratory endpoints in ambulatory participants at 1 year after dosing.

## Results

### Participant disposition

**Fig. 1:**
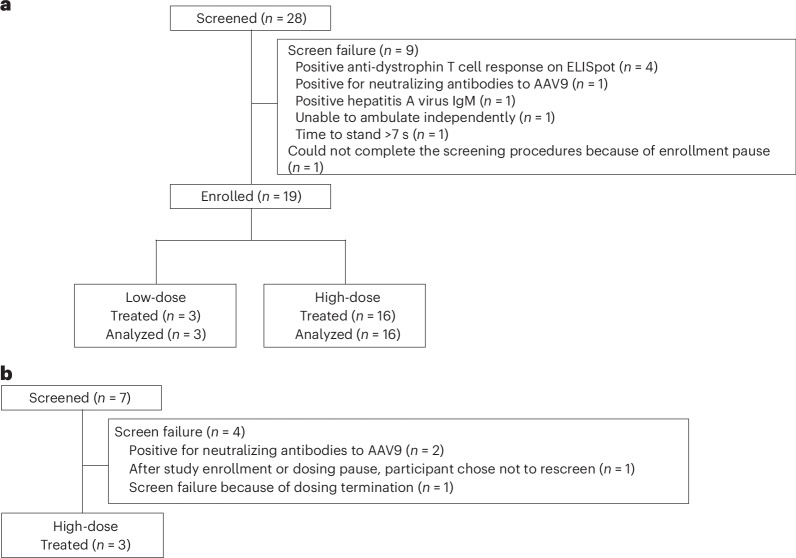
Participant disposition for ambulatory and nonambulatory participants. **a**, Twenty-eight screenings were completed in ambulatory participants with 24 unique ambulatory participants (four participants were rescreened and met the enrollment criteria the second time). Five unique participants did not pass screening and were not assigned to treatment. Participants who met the enrollment criteria on being rescreened included two with positive anti-dystrophin T cell response on enzyme-linked immunosorbent spot (ELISpot) during the initial screen (this exclusion criterion was later removed from the protocol given the absence of myositis and myocarditis observed in this trial): one with positive hepatitis A virus IgM during the initial screen and one who was unable to complete the screening procedures during the first screen because of study enrollment pauses. **b**, Seven nonambulatory participants were screened with 3 enrolled. One participant who was screened twice was only counted once.

The first ambulatory participant in this study was enrolled on 23 January 2018 and the last participant was enrolled on 28 March 2022. Twenty-eight screenings were completed with 24 unique ambulatory participants (four ambulatory participants were rescreened). Five unique participants did not meet eligibility criteria, with one being excluded due to the presence of neutralizing antibodies to AAV9 (other reasons for screen failure are given in Fig. 1a). Nineteen ambulatory participants were assigned to and received the study treatment (low-dose fordadistrogene movaparvovec, *n* = 3; high-dose fordadistrogene movaparvovec, *n* = 16). All ambulatory participants completed ≥1 year of follow-up. Mean ± s.d. age at dosing was 8.6 ± 1.6 years. The mutations for ambulatory participants are summarized in Table 1 and all individual mutations are listed in Supplementary Table 1. Additional demographics and clinical characteristics for the study participants are shown in Table 1. All participants who were enrolled and received treatment had negative results for neutralizing antibodies against AAV9 at baseline; 17 participants were also tested and had negative results for total binding antibodies against AAV9 at baseline.

**Table 1:** Demographics and characteristics of participants treated with fordadistrogene movaparvovec

Characteristic	Ambulatory participants			Nonambulatory participants
	Low-dose fordadistrogene movaparvovec (*n* = 3)	High-dose fordadistrogene movaparvovec (*n* = 16)	All (*n* = 19)	High-dose fordadistrogene movaparvovec (*n* = 3)
Age at dosing (years), mean ± s.d.	8.6 ± 1.0	8.7 ± 1.7	8.6 ± 1.6	15.1 ± 1.0
Age at screening (years), median (range)	8.0 (7–9)	8.0 (6–12)	8.0 (6–12)	14.0 (13–16)
Age category, *n* (%)				
≥4 to <8 years	1 (33.3)	6 (37.5)	7 (36.8)	–
≥8 to ≤12 years	2 (66.7)	10 (62.5)	12 (63.2)	–
Ethnicity (self-reported), *n* (%)				
White	3 (100.0)	13 (81.3)	16 (84.2)	1 (33.3)
Black	0	1 (6.3)	1 (5.3)	1 (33.3)
Asian	0	2 (12.5)	2 (10.5)	–
Not reported	–	–	–	1 (33.3)
Weight at baseline (kg), mean ± s.d.	27.9 ± 8.7	26.1 ± 6.2	26.4 ± 6.4	43.7 ± 3.6
Mutation type, *n* (%)				
Exon deletion	1 (33.3)	7 (43.8)	8 (42.1)	2 (66.7)
Exon duplication	0	3 (18.8)	3 (15.8)	–
Point mutation				1 (33.3)
Indel	1 (33.3)	1 (6.3)	2 (10.5)	–
Nonsense	1 (33.3)	4 (25.0)	5 (26.3)	–
Cryptic splice donor site	0	1 (6.3)	1 (5.3)	–
Glucocorticoid use at screening				
Prednisone/prednisolone				
*n* (%)	1 (33.3)	8 (50.0)	9 (47.4)	2 (66.7)
mg kg^−1^ daily dose, mean ± s.d.	0.40 ± N/A	0.59 ± 0.06	0.57 ± 0.08	0.45 ± 0.05
Duration of use (months), mean ± s.d.	61.3 ± N/A	23.0 ± 14.04	27.3 ± 18.33	28.6 ± 5.00
Deflazacort				
*n* (%)	2 (66.7)	8 (50.0)	10 (52.6)	1 (33.3)
mg kg^−1^ daily dose, mean ± s.d.	0.84 ± 0.2	0.76 ± 0.1	0.77 ± 0.1	0.60 ± N/A
Duration of use (months), mean ± s.d.	36.9 ± 40.2	31.5 ± 29.8	32.5 ± 29.6	34.6 ± N/A

N/A, s.d. not available for *n* = 1.

Seven nonambulatory participants were screened (four participants did not meet the eligibility criteria); three nonambulatory participants all received the high dose of fordadistrogene movaparvovec; Fig. 1b). Mean ± s.d. age at dosing was 15.1 ± 1.0 years. Additional demographics and clinical characteristics for the nonambulatory study participants are shown in Table 1. All participants who were enrolled and received treatment had negative results for neutralizing antibodies against AAV9 at baseline.

The external control cohort was derived from 156 placebo-treated participants from two previous interventional trials of DMD; 40 from Wagner et al.^14^ and 116 from Victor et al.^15^ Of these 156 external study participants, 59 met the eligibility criteria (that is, aged 4–12 years, rise from floor in ≤7 s, ability to walk 10 m unaided, left ventricular ejection fraction (LVEF) ≥55%, stable steroid regimen for ≥6 months at screening), and had nonmissing North Star Ambulatory Assessment (NSAA) total scores at 1 year. This group of 59 formed the external control cohort.

### Primary outcome (adverse events and safety assessments)

#### Ambulatory participants

In this ambulatory population, there were no dose-limiting adverse events (AEs) with the two doses tested in this study. Through 1 year after dosing, 162 treatment-emergent AEs (TEAEs) were reported among 19 participants and 102 events were deemed related to study treatment (Table 2). The most common TEAEs were vomiting (*n* = 15), nausea (*n* = 10), thrombocytopenia (*n* = 9), pyrexia (*n* = 9), decreased appetite (*n* = 8), fatigue (*n* = 7) and headache (*n* = 7). Three TEAEs were considered severe: inguinal hernia in the low-dose group (unrelated to study treatment) and thrombocytopenia and acute kidney injury in the high-dose group (both related to study treatment). One mild TEAE of infusion-site reaction was reported in the high-dose group. Physical examination abnormalities are included in Table 2; of note, there were two cases of affect lability.

**Table 2:** Summary of AEs (up to 1 year) in participants treated with fordadistrogene movaparvovec

Event	Ambulatory participants						Nonambulatory participants	
	Low-dose fordadistrogene movaparvovec (*n* = 3)		High-dose fordadistrogene movaparvovec (*n* = 16)		All (*n* = 19)		High-dose fordadistrogene movaparvovec (*n* = 3)	
	No. events	No. individuals	No. events	No. individuals	No. events	No. individuals	No. events	No. individuals
Any TEAE, *n*	18	3	144	16	162	19	26	3
Any treatment-related TEAE, *n*	12	3	90	15	102	18	15	3
Any severe TEAE, *n*	1	1	2	2	3	3	2	2
Any serious TEAE, *n*	0	0	3	3	3	3	2	2
Thrombocytopenia	0	0	1	1	1	1	0	0
Acute kidney injury	0	0	1	1	1	1	0	0
Dehydration	0	0	1	1	1	1	0	0
Hemolytic uremic syndrome	0	0	0	0	0	0	1	1
Cardiogenic shock	0	0	0	0	0	0	1	1

Common treatment-related TEAEs,^a^ *n* (%)	Low-dose fordadistrogene movaparvovec		High-dose fordadistrogene movaparvovec		All ambulatory participants		Nonambulatory participants	
Anemia	0	2 (12.5)	2 (10.5)	0	0	0	0	0
Thrombocytopenia	0	9 (56.3)	9 (47.4)	0	0	0	0	0
Upper abdominal pain	0	3 (18.8)	3 (15.8)	0	0	0	0	1 (33.3)
Constipation	0	3 (18.8)	3 (15.8)	0	0	0	0	0
Diarrhea	0	3 (18.8)	3 (15.8)	0	0	0	0	0
Nausea	2 (66.7)	8 (50.0)	10 (52.6)	0	0	0	0	3 (100.0)
Vomiting	2 (66.7)	13 (81.3)	15 (78.9)	0	0	0	0	3 (100.0)
Fatigue	1 (33.3)	6 (37.5)	7 (36.8)	0	0	0	0	0
Pyrexia	1 (33.3)	8 (50.0)	9 (47.4)	0	0	0	0	0
Liver injury	0	2 (12.5)	2 (10.5)	0	0	0	0	1 (33.3)
Nasopharyngitis	0	2 (12.5)	2 (10.5)	0	0	0	0	0
Sinusitis	0	3 (18.8)	3 (15.8)	0	0	0	0	0
Fall	0	5 (31.3)	5 (26.3)	0	0	0	0	0
C4 decreased	0	5 (31.3)	5 (26.3)	0	0	0	0	0
GLDH increased	0	3 (18.8)	3 (15.8)	0	0	0	0	0
Decreased appetite	2 (66.7)	6 (37.5)	8 (42.1)	0	0	0	0	0
Dehydration	0	5 (31.3)	5 (26.3)	0	0	0	0	0
Pain in extremity	1 (33.3)	1 (6.3)	2 (10.5)	0	0	0	0	0
Headache	0	7 (43.8)	7 (36.8)	0	0	0	0	3 (100.0)
Affect lability	1 (33.3)	1 (6.3)	2 (10.5)	0	0	0	0	0
Hematuria	0	2 (12.5)	2 (10.5)	0	0	0	0	0
Ketonuria	0	2 (12.5)	2 (10.5)	0	0	0	0	0
Proteinuria	0	2 (12.5)	2 (10.5)	0	0	0	0	0
Cough	0	2 (12.5)	2 (10.5)	0	0	0	0	0
Rash	0	2 (12.5)	2 (10.5)	0	0	0	0	0
Hypertension	0	2 (12.5)	2 (10.5)	0	0	0	0	0
Hemolytic uremic syndrome	0	0	0	0	0	0	0	1 (33.3)
Cardiogenic shock	0	0	0	0	0	0	0	1 (33.3)
Hepatic enzyme increased	0	0	0	0	0	0	0	1 (33.3)
Troponin increased	0	0	0	0	0	0	0	1 (33.3)

^a^Occurring in more than one participant. One participant in the high-dose fordadistrogene movaparvovec group experienced infusion-site reaction; this was deemed treatment-related.

Three serious TEAEs (all deemed treatment-related) were reported in three participants in the high-dose group: thrombocytopenia and acute kidney injury (both described above), and dehydration. Among these, thrombocytopenia and acute kidney injury were consistent with the clinical syndrome of thrombotic microangiopathy (TMA), with each case including reduced complement factor C3 or complement factor C4 levels, reduced platelets, evidence of hemolysis and changes in serum creatinine and cystatin C. After medical management, including 1–2 doses of intravenous eculizumab (600 mg) and platelet transfusion (*n* = 1) or dialysis (*n* = 1), both TEAEs resolved within 15 days. At 1 year after infusion, the mean (±s.d.) absolute change from baseline in LVEF was −0.98% ± 4.3% (95% confidence interval (CI) = –3.5% to 1.5%) for the high-dose ambulatory group, and −0.68% ± 3.2% (95% CI = –8.6% to 7.2%) in the low-dose ambulatory group^16^. Other safety assessments, including abnormal findings from clinical laboratory tests, vital signs and electrocardiograms are shown in Supplementary Tables 2–4.

At baseline, one participant in the high-dose group had experienced suicidal ideation (according to the Columbia-Suicide Severity Rating Scale (C-SSRS)) reported at the baseline visit. This participant was assessed throughout the study and did not experience suicidal ideation.

Liver function was monitored primarily using glutamate dehydrogenase (GLDH) levels because standard tests for aspartate and alanine transaminases are unreliable indicators of liver function in the study population; these enzymes are present in muscle and typically elevated in patients with DMD, can be increased by activity and can be reduced after gene therapy treatment. Five participants had transient increases of GLDH levels greater than 25 U l^−1^ (2.5 times the upper limit of normal (ULN)), with the maximum for each participant ranging from 26 to 54 U l^−1^ reported as *n* = 3 with GLDH increased and *n* = 2 liver injury. Gamma-glutamyl transferase (GGT) rose above the ULN in two of the five cases. Two had an onset in the first 14 days of the study, two between days 27 and 29, and one on day 134. The participant with increased GLDH on day 134 was noted to have discontinued his prednisone for 4 weeks before this event. Two of these five participants with both elevated GLDH and GGT levels required supplemental prednisone. Both participants were treated with a higher daily dose of oral prednisone for 4–5 weeks.

#### Nonambulatory participants

In this nonambulatory population, there were no dose-limiting AEs with the high dose tested in this study. In this nonambulatory group, 26 TEAEs were reported among three participants; 15 AEs were deemed related to study treatment. The most common TEAEs were nausea (*n* = 3), vomiting (*n* = 3) and headache (*n* = 3) (Table 2). One moderate TEAE of liver injury was reported in the nonambulatory cohort. Two TEAEs (deemed treatment-related) were reported in two participants and were considered serious: hemolytic uremic syndrome and fatal cardiogenic shock. A 16-year-old nonambulatory participant with DMD experienced fatal cardiogenic shock 6 days after receiving the high-dose of fordadistrogene movaparvovec; this case is described in detail elsewhere^16,17^.

Physical examination abnormalities are included in Table 2. At 1 year after infusion, the mean (±s.d.) absolute change from baseline in LVEF of the two nonambulatory participants was −3.1% ± 1.8% (95% CI = −19.3% to 13.0%)^16^. Other safety assessments, including abnormal findings from clinical laboratory tests, vital signs and electrocardiograms are shown in Supplementary Tables 2–4. At baseline, no participants experienced suicidal ideation (according to the C-SSRS); however, one participant experienced suicidal ideation at month 6.

### Secondary outcomes

**Fig. 2:**
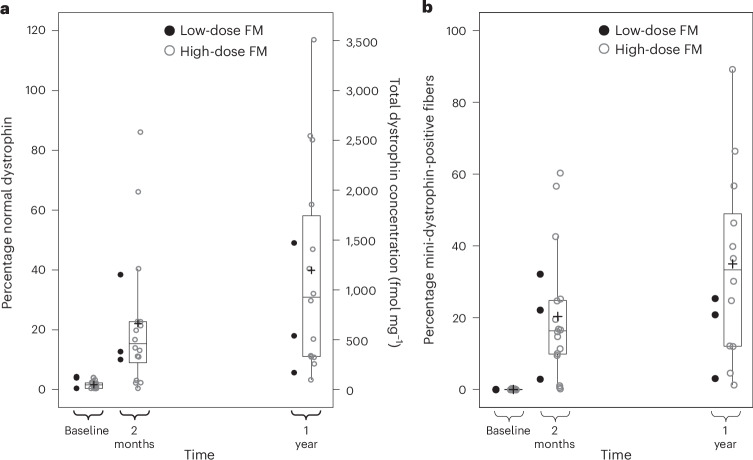
Expression of dystrophin and distribution of mini-dystrophin in muscle biopsy samples from ambulatory participants assessed using LC–MS and automated image analysis of immunofluorescence, respectively. **a**, LC-MS. **b**, Automated image analysis of immunofluorescence. *n* = 3 for low-dose fordadistrogene movaparvovec (FM) and *n* = 16 for high-dose fordadistrogene movaparvovec.

Robust and durable expression and distribution of dystrophin protein was observed in the muscle of the high-dose ambulatory group (Fig. 2a,b). Liquid chromatography–mass spectrometry (LC–MS) was used to detect endogenous dystrophin and mini-dystrophin. Mean (95% CI) dystrophin levels in the high-dose group, expressed as the percentage of normal dystrophin (normalized against the mean of non-dystrophic control tissues; *n* = 20)^18^, were 1.4% (0.8 to 2.0) at baseline (*n* = 16), 21.9% (11.9 to 34.2) at 2 months (*n* = 16) and 39.7% (23.0 to 58.3) at 1 year (*n* = 14). The corresponding mean (95% CI) dystrophin concentration (expressed as fmol mg^−1^ of total protein) was 41.5 (23.9 to 60.8) at baseline, 656.7 (357.01 to 1,026.3) at 2 months and 1,192.3 (690.2 to 1,748.7) at 1 year. The mean (95% CI) percentage of mini-dystrophin-positive fibers, assessed using the automated image analysis of immunofluorescence (Supplementary Fig. 2) was 0.1% (0.1 to 0.2) at baseline (*n* = 16), 20.3% (12.2 to 29.3) at 2 months (*n* = 16) and 34.8% (21.1 to 49.8) at 1 year (*n* = 12). Two biopsy samples could not be included in the 1-year analyses because of unacceptable quality.

**Extended Data Fig. 1:**
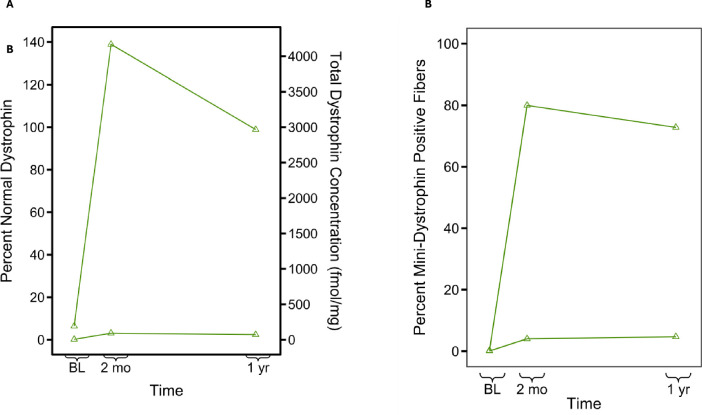
Expression of dystrophin and distribution of mini-dystrophin in muscle biopsy samples from non-ambulatory participants assessed by (A) LC-MS and (B) automated image analysis of immunofluorescence, respectively. In (**A**) LC-MS (n = 2) and (**B**) automated image analysis of immunofluorescence (n = 2). High-dose (green open triangles = mean) groups. BL, baseline; LC-MS, liquid chromatography–mass spectrometry.

Relative to the high-dose group, dystrophin expression levels and the percentage of mini-dystrophin-positive fibers were, on average, numerically lower in the low-dose group (*n* = 3) at 1 year (Fig. 1 and Extended Data Table 1). The mean change in dystrophin and mini-dystrophin from 2 months to 1 year was also lower in the low-dose group versus the high-dose group. The expression levels for nonambulatory participants are shown in Extended Data Fig. 1 and described in Extended Data Table 2.

### Exploratory outcomes

The exploratory functional outcomes for the external control analysis were not prespecified in the original trial protocol, but were defined in the statistical analysis plan before the release of the database for the 1-year analysis. To estimate the relative effect of high-dose fordadistrogene movaparvovec, functional results were compared with the external control cohort described above. The demographics and baseline characteristics were comparable between the two groups (Table 1 and Extended Data Table 3) except for minor differences in baseline rise from floor velocity and 10-m run/walk velocity. The propensity score (PS)-adjusted analysis balanced these differences so that no covariates had a standardized difference greater than 0.1 (Extended Data Table 3).

**Fig. 3:**
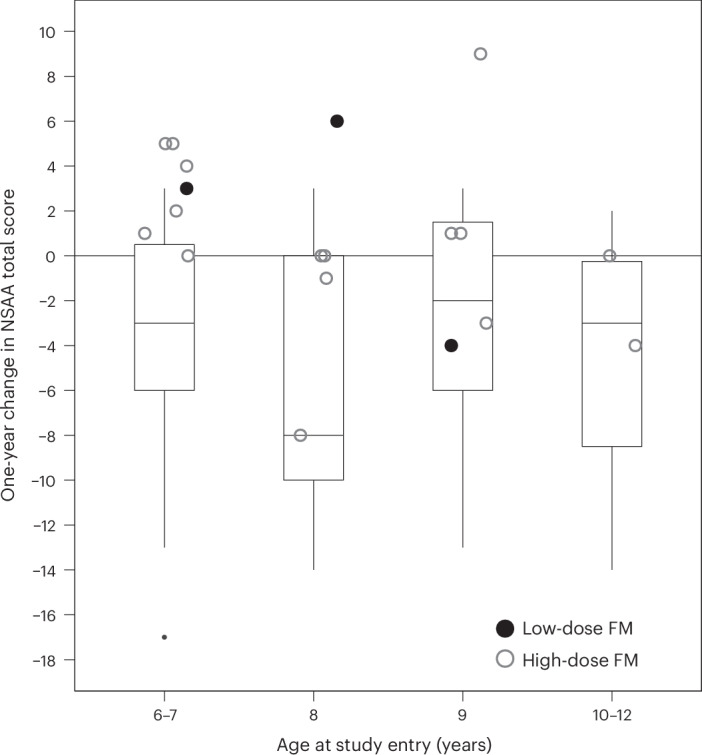
One-year change in NSAA total score in ambulatory participants stratified according to age at study entry. Individual values for participants in the low-dose (filled circles, *n* = 3) and high-dose (open circles, *n* = 16) fordadistrogene movaparvovec (FM) groups of the current study are shown along with box and whisker plots. The box plots display the interquartile range from the 25th to the 75th percentile. The line inside the box indicates the median value. The whiskers indicate the range within 1.5 times the interquartile range, with outliers shown as individual points. The small black dot (bottom left) represents an outlier in the external control cohort. The NSAA total score is based on assessment of 17 ambulatory actions and ranges from 0 to 34, with a higher score indicating greater functional ability. In the external control cohort (*n* = 59), the median NSAA total score at baseline was 28.0 in the 6–7-year-old group (*n* = 23), 28.0 in the 8-year-old group (*n* = 15), 26.0 in the 9-year-old group (*n* = 11) and 24.5 in the 10–12-year-old group (*n* = 10).

**Extended Data Fig. 2:**
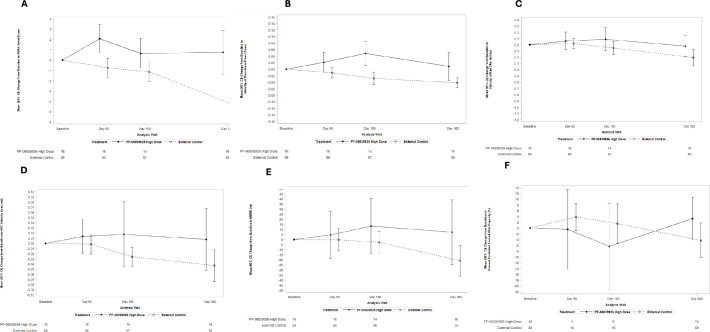
Plots of changes (from baseline to 1 year) in functional endpoints in ambulatory high-dose participants versus external control cohort by visit. The external control cohort was derived from 156 placebo-treated participants from two previous interventional trials of DMD; 40 from Wagner et al., and 116 from Victor et al. Of these 156 external study participants, 59 met the eligibility criteria (ie, ages 4–12 years, rise from floor in ≤7 s, ability to walk 10 meters unaided, left ventricular ejection fraction [LVEF] ≥55%, stable steroid regimen for ≥6 months at screening), and had non-missing North Star Ambulatory Assessment (NSAA) total scores at 1 year. This group of 59 formed the external control cohort. **A**) Mean (95%) change from baseline in NSAA total score; **B**) Mean (95%) change from baseline in velocity of rise from floor; **C**) Mean (95%) change from baseline in velocity of run 10 m; **D**) Mean (95%) change from baseline in 4SC velocity; **E**) Mean (95%) change from baseline in 6MWD, **F**) Mean (95%) change from baseline in percent predicted forced vital capacity. At baseline, n = 16 for high dose and n = 59 for external control, except for panel **F**, n = 12 participants available at baseline in the high dose group. All other n numbers are given below each figure panel. 10 M w/r, time to walk/run 10 meters; 4SC w/r, four-stair climb walk/run; 6MWD, 6-minute walk distance; NSAA, North Star Ambulatory Assessment; SE, standard error.

Mean (s.e.) changes from baseline to 1 year in functional endpoints, before inverse probability treatment weighting (IPTW-ATT), are presented in Extended Data Fig. 2. Improvement in the NSAA total score at 1 year was observed in participants receiving high-dose fordadistrogene movaparvovec and was significantly different from the decline in function observed in the external control cohort (mean (s.d.) 0.8 (4.0) versus −4.2 (5.3) (*P* = 0.0026)) (Fig. 3 and Table 3). After the IPTW-ATT adjustment for potential imbalance described in Extended Data Table 3, the mean ± s.e. was 0.8 ± 1.0 for high-dose fordadistrogene movaparvovec versus −2.7 ± 0.8 for the external control cohort; the difference between the two groups in the change from baseline in NSAA total score was 3.4 (95% CI = 0.9 to 5.8; one-sided *P* = 0.0026) (Table 3).

**Table 3:** Change (from baseline to 1 year) in functional assessments in ambulatory participants and in the external control cohort

Outcome measure	fordadistrogene movaparvovec			External cohort (*n* = 59)	High-dose versus external cohort	
	Low-dose (*n* = 3)	High-dose (*n* = 16)	All (*n* = 19)		Mean difference (95% CI)^a^	One-sided *P*
NSAA total score						
Mean/median score at baseline	26.3/25.0	25.8/27.0	25.8/27.0	26.0/27.0	–	–
Change at 1 year, mean ± s.d.	+1.7 ± 5.1	+0.8 ± 4.0	+0.9 ± 4.0	−4.2 ± 5.3	–	–
Change at 1 year, median	+3.0	+0.5	+1.0	−3.0	–	–
IPTW-ATT						
Change at 1 year, LS mean (s.e.)	–	0.8 (0.97)	–	−2.7 (0.77)	3.4 (0.9 to 5.8)	0.0026
Rise from floor velocity, 1 s^−1^						
Mean/median velocity at baseline	0.31/0.32	0.28/0.30	0.29/0.31	0.22/0.20	–	–
Change at 1 year, mean ± s.d.	+0.01 ± 0.12	+0.01 ± 0.08	+0.01 ± 0.08	−0.04 ± 0.06	–	–
Change at 1 year, median	+0.05	0	+0.01	−0.04	–	–
IPTW-ATT						
Change at 1 year, LS mean (s.e.)	–	0.01 (0.02)	–	−0.06 (0.01)	0.07 (0.03 to 0.12)	0.0007
10-m w/r velocity, m s^−1^						
Mean/median velocity at baseline	2.44 / 2.56	2.29 / 2.20	2.31 / 2.22	1.96 / 1.96	–	–
Change at 1 year, mean ± s.d.	−0.03 ± 0.24	−0.02 ± 0.32	−0.03 ± 0.31	−0.21 ± 0.51	–	–
Change at 1 year, median	−0.04	0.015	0	−0.16	–	–
IPTW-ATT						
Change at 1 year, LS mean (s.e.)		−0.02 (0.08)		−0.19 (0.08)	0.17 (−0.05 to 0.39)	0.0606
4SC velocity, 1 s^−1^						
Mean/median velocity at baseline	0.36/0.33	0.37/0.35	0.37/0.33	0.31/0.29	–	–
Change at 1 year, mean ± s.d.	+0.06 ± 0.11	+0.01 ± 0.11	+0.02 ± 0.11	−0.04 ± 0.12	–	–
Change at 1 year, median	0	−0.02	−0.02	−0.06	–	–
IPTW-ATT						
Change at 1 year, LS mean (s.e.)		0.01 (0.03)		−0.01 (0.03)	0.02 (−0.07 to 0.10)	0.3019
6MWD, m						
Mean/median distance at baseline	432.3/414.0	386.0/394.5	393.3/396.0	380.0/375.0	–	–
Change at 1 year, mean ± s.d.	+10.7 ± 94.9	+7.3 ± 59.9	+7.8 ± 63.2	−20.8 ± 58.4	–	–
Change at 1 year, median	−29.0	+3.0	−1.0	−23.0	–	–
IPTW-ATT						
Change at 1 year, LS mean (s.e.)		7.3 (14.7)		−10.6 (10.5)	17.8 (−19.6 to 52.1)	0.1540
PUL 2.0						
Mean/median score at baseline	38.3/39.0	39.4/40.0	39.2/40.0	37.4/38.0	–	–
Change at 1 year, mean ± s.d.	+1.3 ± 1.5	+0.9 ± 2.5	+0.9 ± 2.3	−0.3 ± 1.62	–	–
Change at 1 year, median	+1.0	+1.0	+1.0	0	–	–
% pFVC						
Mean/median score at baseline	94.0/84.0	95.6/96.0	95.3/96.0	107.4/ 04.0	–	–
Change at 1 year, mean ± s.d.	+17.3 ± 27.2	+3.3 ± 11.5	+6.4 ± 16.1	−4.3 ± 23.1	–	–
Change at 1 year, median	+8.0	+4.0	+6.0	−2.1	–	–
IPTW-ATT						
Change at 1 year, LS mean (s.e.)		3.3 (3.2)		−7.8 (5.0)	11.0 (−0.3 to 23.0)	0.0295

IPTW-ATT analysis was performed using a linear outcome model to estimate the average treatment effect among the treated. PS weighting was applied to balance the observed covariates between the high-dose fordadistrogene movaparvovecand external control groups. Bootstrap resampling was used to estimate the s.e. for the treatment effect and to construct the 95% CI. The *P* value was calculated as the proportion of bootstrap samples with a mean difference more extreme than the observed value, based on the empirical bootstrap distribution. The 95% CIs and *P* values are presented for descriptive purposes, with no adjustments made for multiple comparisons. ^a^Bootstrap standard error: bootstrap-bias-corrected 95% CI. 10-m w/r, time to walk/run 10 m; 4SC w/r, four-stair climb walk/run; 6MWD, 6-min walk distance; LS, least squares; pFVC, predicted forced vital capacity; PUL, performance of the upper limb.

Changes in the NSAA total score at 1 year varied according to age (Extended Data Table 4 and Extended Data Fig. 2). In the high-dose fordadistrogene movaparvovec group, mean ± s.d. change in NSAA total score was 2.8 ± 0.8 and −0.5 ± 1.3 in participants aged 6–7 years (*n* = 6) and 8–12 years (*n* = 10), respectively. The difference between the high-dose fordadistrogene movaparvovec and external control groups was 5.1 (95% CI = 2.3 to 7.9) for participants aged 6–7 years and 2.8 (95% CI = − 0.4 to 6.2) for participants aged 8–12 years.

The NSAA was also analyzed considering each of the 17 individual NSAA items as a unique skill that could be gained, improved or maintained. A ‘skill gained’ was defined as one that could not be performed at baseline (score = 0) but could be performed (score = 1 or 2) at 1 year. Seven of 16 study participants and 17 of 59 external cohort participants had at least one item with score = 0 at baseline. Of these, 5 of 7 (71.4%) study participants had at least one skill gained compared with 5 of 17 (29.4%) in the external cohort. A ‘skill improved or maintained’ was defined as one that had the same or higher nonzero score at 1 year compared with baseline. The median number of skills improved or maintained among study participants was 16.0 versus 12.0 in the external control cohort.

There was a consistent trend toward maintenance or improvement in other exploratory measures of ambulatory, upper-limb and respiratory function in fordadistrogene movaparvovec-treated participants compared with the external control cohort (Table 3 and Extended Data Fig. 2).

### Sensitivity analysis

Sensitivity analysis of change from baseline in the NSAA total score at 1 year using doubly robust methods showed similar results (adjusted mean difference = 3.6; 95% CI = 0.8 to 8.8).

## Discussion

Gene therapy is a promising treatment for DMD, given the unmet need for safe and effective therapies^6^. This first-in-human and first-in-patient study demonstrated that a single intravenous infusion of fordadistrogene movaparvovec had an acceptable safety profile in ambulatory participants for at least 1 year after treatment. This conclusion is supported by the findings that all serious treatment-related TEAEs (thrombocytopenia, acute kidney injury and dehydration) resolved within 15 days of onset and that most TEAEs were mild to moderate in severity over the 1-year follow-up. Two of the serious TEAEs were consistent with TMA, which has also been associated with other AAV-based gene therapies^19–24^. It is hypothesized that anti-AAV antibodies, binding to circulating AAV vector to form immune complexes that activate endothelial and complement pathways, trigger TMA^20^. Participants with TMA were successfully managed with supportive care, eculizumab, hemodialysis or platelet transfusion. A subsequent protocol amendment increasing the post-gene therapy regimen of glucocorticoids was effective in reducing the risk of TMA for the additional ambulatory participants.

Although not observed in this clinical trial, three cases of transient myositis (two of which were associated with myocardial inflammation) were observed roughly 4 weeks after infusion of fordadistrogene movaparvovec; similar manifestations have been associated with other miniaturized dystrophin gene therapy candidates and are related to a T cell-mediated response against the hinge 1 or spectrin repeat 1 protein domains of dystrophin^25^. Ongoing miniaturized dystrophin gene therapy trials now contain exclusions for deletion mutations that encode these domains^26–28^.

Like the fatal event that occurred in the nonambulatory participant in this trial, there was a recent case of fatality in a 27-year-old patient with DMD shortly after receiving a high dose of gene therapy^29^. That patient was treated with AAV9 encapsidating an inactivated *Cas9* transgene with the goal of upregulating an alternate full-length dystrophin variant. The patient experienced cardiac arrest 6 days after infusion and died of respiratory failure 2 days later, with severe diffuse alveolar damage noted postmortem. These two cases suggest that older patients with DMD may have less ability to cope with AAV-induced immune responses and downstream consequences.

Dystrophin replacement strategies require widespread distribution of protein expression in skeletal and cardiac tissue in sufficient concentrations to compensate for the lack of functional dystrophin in patients with DMD. In this phase 1b study, robust expression of mini-dystrophin was evident in muscle biopsy samples from participants treated with a high dose of fordadistrogene movaparvovec. Mean protein levels and the proportion of mini-dystrophin-positive fibers were substantially increased at 2 months and further elevated at 1 year. The normalized dystrophin levels observed in most study participants are in the range associated with patients who have a milder disease course^2,18,30^. Expression in a high percentage of fibers is particularly noteworthy because this may be a key factor contributing to integrated muscle function. It is currently unclear whether the addition of miniaturized dystrophin will mitigate disease progression as effectively as the somewhat larger proteins expressed in patients with Becker muscular dystrophy, particularly because some dystrophic changes will have occurred before gene therapy.

Given the open-label, nonrandomized nature of this study and ongoing research with gene therapy in DMD, comparisons with external control data sources are of interest to contextualize the benefit of gene therapy. Several guidance documents, including International Council for Harmonisation of Technical Requirements for Pharmaceuticals for Human Use topic E10, highlight that a major limitation in the use of external controls is the inability to control bias^31^. To reduce the impact of those limitations, participants in the external control cohort were selected from the two contemporary randomized controlled trials because they more closely aligned with the Pocock criteria^32^ for evaluating external data sources. Furthermore, the external control cohort was derived to have age, function, ambulation, cardiac function and steroid use similar to those of the study population. The mean daily dose of deflazacort at baseline differed slightly among the high-dose study participants (*n* = 8; 0.76 mg kg day^−1^) compared with those in the external control cohort (*n* = 30; 0.67 mg kg day^−1^); however, this difference is not expected to have affected functional outcomes over a 1-year period.

The PS-adjusted analysis showed consistently favorable results toward improvement, although this varied somewhat according to screening age in measures of motor and respiratory function among fordadistrogene movaparvovec-treated participants compared with an external control cohort of placebo-treated participants. Importantly, participants treated with high-dose fordadistrogene movaparvovec exhibited an adjusted mean improvement (from baseline to 1 year) in NSAA total score of 0.8 points compared with a decline of 2.7 points in the external control cohort (treatment difference of 3.4 points). For high-dose fordadistrogene movaparvovec-treated participants aged 6–7 years, an even larger adjusted mean increase (2.8 points) and a larger adjusted treatment difference (5.1 points) were observed. A greater proportion of treated participants gained at least one NSAA skill compared with the external control cohort; the median number of improved or maintained skills was 16.0 in the study participants compared with 12.0 in the external control cohort.

The NSAA provides a reliable, validated and clinically relevant measure of functional motor ability (and thus disease progression) in ambulatory children with DMD^33,34^. Maintenance or improvement in function is particularly meaningful given the age of the participants in this study. Overall, these findings suggest that expression of mini-dystrophin produced the desired effect, in which functional capacities are mostly stabilized or improved.

Limitations of this study included the small sample size (particularly the nonambulatory cohort), the open-label design (which may introduce bias into NSAA evaluations because clinical evaluators knew that patients had been treated) and the use of an external control cohort as a comparator for the exploratory functional endpoints not prespecified in the protocol. Although the potential for selection bias was minimized by the stringent inclusion criteria applied to the external control cohort and by adjusting for known prognostic factors in the PS-adjusted analysis, residual confounding may still remain and be difficult to control for, particularly in datasets where differences in important prognostic factors are unknown or not measured in a single dataset. One of the advantages of using IPTW-ATT for baseline covariate adjustment is that it includes all participants in small studies weighted by the PS to mitigate confounding. However, the limited number of treated participants restricts the inclusion of all possible confounders in the PS model and requires the use of a simple linear term to limit PS model overparameterization that can affect estimation if the model is misspecified. An increase in sample size would enable a more robust estimation, especially in the analysis stratified according to age.

The preliminary results presented for this ambulatory population indicated that fordadistrogene movaparvovec had an acceptable safety profile in ambulatory participants. Furthermore, these findings indicated the potential for benefit by slowing or preventing loss of function. Overall, these findings supported continuing clinical development of this investigational gene therapy. Dosing in a phase 3 placebo-controlled study (ClinicalTrials.gov registration no. NCT04281485) in participants aged 4–7 years with DMD was paused on 7 May 2024 because of a fatal serious AE in a phase 2 trial (ClinicalTrials.gov registration no. NCT05429372) in participants aged 2–3 years with DMD. Furthermore, on 12 June 2024, Pfizer announced topline results from the phase 3 study, which showed no significant difference in the primary endpoint (change from baseline in NSAA) and key secondary endpoints (10-m run/walk velocity and time to rise from floor velocity) between the fordadistrogene movaparvovec and placebo groups^35^. Therefore, further clinical development of fordadistrogene movaparvovec has been discontinued.

## Methods

### Study design and oversight

This ongoing, nonrandomized, open-label, ascending-dose, phase 1b study (ClinicalTrials.gov registration no. NCT03362502) was initiated in January 2018 at three sites in the United States (Supplementary Fig. 3 and detailed in the Supplementary Protocol) and was conducted in compliance with the ethical principles of the Declaration of Helsinki 2013 and all International Conference on Harmonisation Good Clinical Practice Guidelines. The protocol was approved by the relevant institutional review board (IRB) or independent ethics committee at each study site (University of Utah IRB, Duke University Health System and UCLA Medical IRB). All participants (or parent or legal guardian) provided written informed consent. Data collection occurred at each study site (study start date 23 January 2018; primary completion date 28 March 2022, ClinicalTrials.gov registration no. NCT03362502). Participants from three sites in the United States were enrolled in the trial.

### Key inclusion and exclusion criteria

Personally signed and dated informed consent or assent (where appropriate) documents were obtained, indicating that the participant or a legally acceptable representative, parent(s) or legal guardian was informed of all pertinent aspects of the study and were willing and able to comply with scheduled visits, treatment plans, laboratory tests and all other required study procedures. Participants were not compensated.

Eligible ambulatory males were aged 4–12 years (inclusive), had a body weight of 15–50 kg (inclusive), had a diagnosis of DMD (confirmed by medical history and genetic testing before screening), were able to tolerate magnetic resonance imaging and muscle biopsies under anesthesia without sedation, with no contraindications to these procedures. Participants were receiving daily glucocorticoids for 6 or more months (stable regimen for 3 or more months before baseline). Ambulatory status was defined as able to walk 10 m unassisted and rise from the floor within 7 s.

Eligible nonambulatory male participants were of any age (ambulation before the participant’s 17th birthday), had a body weight of 75 kg or lower, had a diagnosis of DMD (confirmed by medical history and genetic testing before screening) and were receiving daily glucocorticoids for 6 or more months (stable regimen for 3 or more months before baseline). Nonambulatory status was defined as unable to walk 10 m unassisted and rise from the floor within 7 s, with adequate respiratory function (percentage predicted forced vital capacity (FVC) greater than 40%), adequate upper-limb function (v.2.0), and an entry item A score of 3 or more.

All participants with neutralizing antibodies against the AAV9 capsid at screening were excluded. Additional exclusion criteria included receipt of a live attenuated vaccination (within 3 months) or exposure to an influenza vaccination or systemic antiviral or interferon therapy (within 30 days) before receiving the study treatment; current exposure to systemic immunosuppressant agents other than glucocorticoids; genetic abnormalities in the dystrophin gene as confirmed by the investigator based on the review of DMD genetic testing, that is, any mutation (deletion, duplication, insertion or point mutation) affecting any exon between exons 9 and 13 inclusive; or deletion affecting both exons 29 and 30; prior gene therapy treatment (any therapy introducing exogenous DNA or intended to permanently alter endogenous DNA); gene therapy (other than the study drug); exposure within 6 months before screening (visit 1) to any treatment designed to increase dystrophin expression (including, but not limited to exon-skipping agents and nonsense read-through). These treatments were also prohibited during the period between screening (visit 1) and day 1 (visit 3) and for the first year of the study; participation in other studies involving investigational drug(s) within 30 days or five half-lives, whichever was longer, before the baseline visit; known hypersensitivity to any of the components of the study drug or solution for infusion, such as hypersensitivity to albumin or a diagnosis of (or symptoms suggestive of) hereditary fructose intolerance; presence or history of other musculoskeletal or neurological diseases in addition to DMD; evidence or history of clinically significant hematological, renal, endocrine, pulmonary, gastrointestinal, cardiovascular, hepatic, cancer, autoimmune or allergic disease (including drug allergies, but excluding untreated, asymptomatic, seasonal allergies at the time of dosing); LVEF lower than 55% (LVEF lower than 35% for nonambulatory participants), specified abnormalities in hematology or chemistry tests (absolute neutrophil count less than 1,000 cells per mm^3^; cystatin C greater than 1.2 times ULN; platelets lower than 150 × 10^3^ µl^−1^); positive hepatitis A virus IgM; and hepatitis B surface antigen or hepatitis C antibody. Additional exclusion criteria were serum markers indicating possible autoimmune-mediated hepatitis: antinuclear antibody titer greater than 1:160; total IgG more than two times the ULN; markers of hepatic inflammation or overt or occult cirrhosis as evidenced by one or more of the following: total bilirubin more than 1.5 times the ULN and direct bilirubin ≥0.5 mg dl^−1^; GGT test more than 1.5 times the ULN; prothrombin greater than the ULN; prolonged international normalized ratio greater than the ULN; and GLDH more than two times the ULN. Participants with clinically significant infection within 30 days before study drug administration, and acute or chronic medical or psychiatric conditions, including recent (within the past year) or active suicidal ideation or behavior (within the past 6 months), or laboratory abnormality that may increase the risk associated with study participation or investigational product administration or may interfere with the interpretation of the study results were also excluded. In addition, nonambulatory participants were excluded if there was any injury that may have impacted functional testing. Previous injuries must have fully healed before consenting. For ambulatory participants, prior lower-limb fractures must have fully healed and at least 3 months from the injury date.

### Study treatment

All ambulatory participants received a single intravenous infusion of low-dose (1 × 10^14^ vector genomes per kilogram of body weight; 1 × 10^14^ vg kg^−1^) or high-dose (3 × 10^14^ vg kg^−1^) fordadistrogene movaparvovec, according to dose escalation rules (Methods). Information on the vector and transgene has been described previously^25,36^. All nonambulatory participants received a single intravenous infusion of high-dose fordadistrogene movaparvovec. Vector genome concentration was initially determined via an inverted terminal repeat assay and later transitioned to a transgene-based quantitative PCR assay. For the high dose, 3 × 10^14^ vg kg^−1^ using the inverted terminal repeat assay is approximately equivalent to 2 × 10^14^ vg kg^−1^ using the transgene-based assay.

To mitigate the risk of immune responses, participants were administered a prespecified glucocorticoid regimen during the first 3 months (Methods). After 3 months, participants resumed their baseline glucocorticoid regimen and continued this regimen throughout the first year of follow-up, except for adjustments due to AEs or changes in body weight. Details on the use of concomitant medication, including rescue medication to manage immune responses, are provided in the Methods.

### Endpoints and assessments

The primary endpoints were dose-limiting AEs, and safety and tolerability through 1 year after treatment based on the incidence, severity and causal relationship of TEAEs. These were coded using the Medical Dictionary for Regulatory Activities v.24.0, with severity and relationship to treatment determined by the site investigators. Other safety assessments included the incidence and magnitude of abnormal findings from clinical laboratory tests, physical and neurological examinations, electrocardiograms, LVEF (assessed using magnetic resonance imaging or echocardiogram) and C-SSRS, which were conducted throughout the study through to week 52 (Supplementary Fig. 3).

The secondary endpoint was expression of mini-dystrophin in biceps brachii muscle biopsy samples. Total dystrophin concentration LC–MS and the proportion of mini-dystrophin-positive fibers (assessed using automated image analysis of immunofluorescence) were determined at baseline, 2 months and 1 year after treatment (Methods). The LC–MS assay used for the assessment recognized both full-length dystrophin and mini-dystrophin, while the immunofluorescence assay used a mini-dystrophin-specific antibody; thus, it detects the mini-dystrophin transgene only. The term ‘dystrophin’ is used to describe both forms of the protein.

### Assessment of dystrophin and mini-dystrophin using LC–MS and immunofluorescence

The LC–MS assay used for the quantification of dystrophin and transgene-expressed mini-dystrophin consisted of the following steps: (1) skeletal muscle homogenization in a sodium dodecyl sulfate containing lysis buffer; (2) protein precipitation using acetonitrile; (3) digestion with trypsin to yield the surrogate peptides used for quantification: (4) enrichment of these peptides using antipeptide antibodies; and nanoflow LC coupled to a triple quadrupole mass spectrometer operating in multiple reaction monitoring mode. Stable isotope labeling by amino acids in cell culture mini-dystrophin was used as an internal standard and calibration of the assay was achieved using a mini-dystrophin recombinant protein. The assay was validated as described previously^14^.

The mini-dystrophin/laminin wet chemistry, and image analysis, assays were fully validated before use in this study (Flagship Biosciences). Briefly, slides were fluorescently labeled with mini-dystrophin/laminin and scanned on a 3DHISTECH Pannoramic SCAN II fluorescence scanner in the green and red channels at ×20. Manual annotations were applied across the entire tissue to identify regions of analysis for each specimen to be scored by image analysis with inclusion annotations to capture relevant target tissue (ice, muscle fibers) and exclusion annotations to remove regions on the slide image containing either unanalyzable tissue (for example, necrosis, folding, dust, crush artifacts, other tissue-specific artifacts) or analyzable tissue that was not of interest. The Flagship Biosciences proprietary image analysis algorithm was used for classification and quantification of the staining intensity of the background-corrected red channel mini-dystrophin (representing mini-dystrophin) in each fiber identified by the developed algorithm. The algorithm collected various endpoint data on each fiber in the biopsy. The primary data reported for this study were derived from: (1) the total number of fibers analyzed on the slide; (2) the number of mini-dystrophin-positive fibers; and (3) the mean stain density (MSD) of mini-dystrophin labeling for all fibers. A global threshold for calculating the percentage of mini-dystrophin-positive fibers was established based on all baseline samples from the 19 ambulatory participants. The quantification of the immunofluorescence intensity level (MSD) associated with the individual muscle fibers from these 19 participants was used to calculate the 99th percentile of the overall MSD distribution. The corresponding MSD threshold value was determined. Any muscle fiber with an MSD value above this calculated threshold was considered as a positive fiber. The antibodies, controls and concentrations used for immunofluorescence are shown in Supplementary Table 5. Two sections from each muscle biopsy were analyzed for immunofluorescence; the average results from the two sections were reported per visit per participant.

Prespecified exploratory functional endpoints included change from baseline to 1 year in the NSAA total score^34^, time to rise from floor, time to climb four stairs, 6-min walk distance, time to walk/run 10 m (10-m w/r), performance of the upper limb 2.0 (ref. ^37^) and percentage predicted FVC (%pFVC). Refinement of two exploratory endpoints were made: the number of NSAA items (that is, skills) gained (among participants with at least one baseline individual item score of zero) and the number of skills maintained or improved (among all participants). Patient-reported and parent-reported assessments (as measured using the Pediatric Outcomes Data Collection Instrument assessment) were also included and have been described separately^38^.

### Major protocol amendments relevant to the ambulatory population reported in this study

The protocol and amendments were approved by the IRB and independent ethics committee for each site (University of Utah IRB, Duke University Health System and UCLA Medical IRB).

Amendment 1 detailed the prohibition of influenza vaccine within 30 days before and 3 months after the administration of fordadistrogene movaparvovec, and allowed for flexibility of weight-based calculations for required glucocorticoids to align with standard of care.

Amendment 2 detailed the requirement for observation at the site after administration of fordadistrogene movaparvovec until any observed events had been resolved residing near the investigational site for at least 1 week for ease of follow-up and allowed for medications to treat or prevent side effects, such as antiemetics or antihistamines for nausea and vomiting.

Amendment 3 detailed the requirement to reside near the investigational site for at least 2 weeks, and an additional visit on days 10 and 21 (added to days 4, 7 and 14) for clinical and laboratory assessments. This amendment also detailed the addition of triplicate electrocardiogram at weeks 2 and 4, safety labs added through week 8 to monitor for activation of the complement system and associated clinical findings (including blood smear for schistocytes, haptoglobin and exploratory immune biomarkers) and requirement for eculizumab to be accessible by the investigational sites for use if complement activation was observed. In addition, Amendment 3 detailed the requirement for meningococcal vaccination (at least 2 weeks before dosing) or, if vaccination was contraindicated, prophylactic antibiotics for meningococcus if eculizumab was administered, and allowed for flexibility in timing of after treatment muscle biopsies (that is, at either 2 or 3 months and either 6 or 12 months; note that a single participant underwent a posttreatment biopsy at 3 instead of 2 months; all planned second posttreatment biopsies were performed within 1 month of the 12-month visit).

Amendment 4 detailed the number of enrolled individuals to be increased from 12 to 24, removed the exclusion criterion pertaining to a preexisting T cell response on ELISpot assay, the addition of possible local laboratory samples to be collected and tested on study days 7–10 and extension of complement biomarker collection through week 8. This amendment also detailed the assessment of drug concentrations of fordadistrogene movaparvovec changed to the transgene method, per regulatory request, and increased flexibility in the dosage of protocol-required glucocorticoids just before (that is, intravenous methylprednisolone) and immediately after (that is, oral prednisone) administration of fordadistrogene movaparvovec.

Amendment 5 detailed the requirement for local laboratory testing on days 5–9, and removed the anti-smooth muscle antibody assay and anti-liver kidney microsomal antibody type 1 laboratory tests for autoimmune-mediated hepatitis as exclusion criteria, given the lack of observed hepatic injury associated with fordadistrogene movaparvovec. This amendment also detailed the increased dose of intravenous methylprednisolone from ≥1 to ≥2 mg kg^−1^, increased the dose of oral prednisone/prednisolone from ≥1 mg kg day^−1^ to ≥2 mg kg day^−1^ for the first 2 weeks after administration of fordadistrogene movaparvovec and included all types of vaccinations (such as those potentially available for severe acute respiratory syndrome coronavirus 2) that are prohibited 30 days before and 3 months after the administration of fordadistrogene movaparvovec.

### Staggered dosing design

To mitigate unanticipated risks to participant safety, enrollment was staggered within and between the fordadistrogene movaparvovec (PF-06939926) low-dose and high-dose groups and included a formal review by an external data monitoring committee (EDMC) before dose progression and in the event of any possible safety signals. In each of the dose-level groups, the dosing interval between the first and second participants was at least 6 weeks. If no safety concerns were identified 3 weeks after the second participant was infused, dosing proceeded at ≥3-week intervals. Similarly, if no stopping or pause criteria were met after dosing six consecutive participants at the high-dose level, dosing proceeded at that same dose level at ≥1-week intervals.

For both dose groups, enrollment was paused to accommodate EDMC review if any of the following occurred: any potentially treatment-related serious AE; similar clinically significant safety findings in 50% or more participants at a given dose level (indicating dose-limiting intolerance); repeated alkaline phosphatase or total bilirubin more than two times the ULN; repeated GLDH more than 2.5 times the ULN; a clinical diagnosis of myositis or myocarditis; other findings that, at the discretion of the sponsor study team, investigator or EDMC, indicated that dose escalation should be halted or that dose de-escalation would be appropriate.

In addition to the review of safety data, the 2-month muscle biopsy findings of the three enrolled participants in the low-dose group were reviewed by the EDMC before progressing to dosing of the high-dose group.

### Administration of fordadistrogene movaparvovec

Fordadistrogene movaparvovec was supplied as a sterile frozen solution (5-ml volume) in a clear, single-use vial. The solution was thawed, diluted (in a 250–500-ml solution containing 1.25% serum albumin) and administered intravenously over approximately 2–4 h to participants by qualified site staff (for example, physician, nurse or pharmacist). Participants were monitored throughout infusion and for at least 24 h after infusion completion.

### Glucocorticoid regimen

To mitigate the risk of an immune response and drug-induced liver injury, each participant was administered a prespecified glucocorticoid regimen over the first 3 months of the study. The regimen was revised in the protocol amendments (described above) and is summarized across all participants, including as a single intravenous dose of methylprednisolone (1–2 mg kg^−1^) 1–4 h before receiving the fordadistrogene movaparvovec infusion and daily oral prednisone or prednisolone after treatment (1–2 mg kg^−1^ for the first 2–4 weeks, tapering to 1.0 mg kg^−1^ through the end of the first month, 0.75 mg kg^−1^ for the second month and the higher dose of 0.50 mg kg^−1^ or the pre-study dose for the third month). After 3 months, participants resumed their baseline glucocorticoid regimen and remained on this regimen throughout the first year of follow-up, except for adjustments due to side effects or changes in body weight.

### Use of concomitant medications

Participants could receive angiotensin-converting enzyme inhibitors, beta-blockers, angiotensin II receptor blockers and aldosterone blocker or thiazide diuretics if initiated at least 3 months before screening and stable dosing was planned during the study. Antiemetics were allowed in the first 7–10 days after dosing of fordadistrogene movaparvovec. Other permitted medications included dietary supplements and bisphosphonates.

Medications prohibited throughout the study included immunosuppressant agents (other than glucocorticoids) unless administered in response to immunological reaction, systemic antiviral or interferon therapy unless administered to treat an acute viral infection, other investigational therapies, other gene therapy agents and sedatives for imaging assessments. Treatments aiming to increase dystrophin expression were prohibited from screening through 1 year after treatment. Receipt of vaccinations (including influenza, live attenuated vaccines, mRNA-based or DNA-based vaccines, or nonreplicating viral vaccines) was prohibited from screening through 3 months after treatment.

### Rescue medication

In the event of an infusion-site reaction during the infusion period, administration of treatment could be paused and supportive therapy could be provided according to standard of care (for example, antihistamine therapy). In the event of elevated liver enzymes during the study, glucocorticoid doses could be increased and immunomodulatory treatment could be added as needed after consultation with immunopharmacological or hepatic experts. In response to an AE due to apparent activation of the complement system (for example, abnormality in complement biomarkers, blood smear indicative of hemolysis or more than two consecutive reductions in platelet count), participants could receive at least one dose of the complement inhibitor eculizumab. As eculizumab increases the risk of contracting meningococcal infections, all previously unvaccinated participants were required to receive at least one dose of a meningococcal vaccination at least 30 days before receiving fordadistrogene movaparvovec. In case of vaccine allergy, prophylactic antibiotics for meningococcus were provided along with eculizumab.

### Derivation and analysis of the external control cohort

The responsibility of the internal independent team was to (1) develop the key criteria for the selection of appropriate external control participants from the identified data sources; (2) identify the external control data, including detailed assessment of sources of potential bias and confounders; and (3) identify key covariates to include in the PS analyses.

Evaluation of comparability between the external control data sources and the current study were based on Pocock’s criteria of exchangeability^4^. Based on the independent team’s evaluation, the following criteria (evaluated at baseline) were used for the selection of the external control cohort: (1) aged 4–12 years at screening, inclusive; (2) an ability to ambulate independently at screening (or baseline if screening was missing), defined as a score of 1 or 2 on NSAA item 17 (10-m run/walk), or a score of 0 (not missing data) on NSAA item 17, or a score of 0 (missing data) for NSAA item 17 with a score of 1 or 2 for NSAA item 2 (walk); (3) an ability to rise from the floor within 7 s (as part of the NSAA) at screening (or baseline if screening was missing); (4) LVEF greater than 55% at screening (or at baseline if screening was missing); and (5) nonmissing NSAA total score at baseline (or screening if baseline was missing).

PS analysis was used to adjust for any remaining potential imbalance in baseline demographic and clinical characteristics between the treatment group and the external control cohort. The PS is the probability of treatment assignment conditional on observed baseline characteristics. It allows the analysis of an observational (nonrandomized) study in a way that mimics some of the particular characteristics of a randomized controlled trial^5^. PS were estimated using a multiple logistic regression model that incorporated potential treatment predictors as independent variables and treatment group as a dependent variable (fordadistrogene movaparvovec versus external control cohort). Covariates in the logistic regression model included the following baseline characteristics: age; NSAA total score; rise from floor velocity (/s); and 10-m run/walk velocity (m s^−1^).

In the estimation of the treatment effects, PS were used to adjust for differences between fordadistrogene movaparvovec-treated and external control participants using IPTW-ATT among treated participants. The IPTW-ATT weighting was calculated as follows:

$$\begin{array}{l}{Wi}=1\,{\text{if the}}\,{i}^{{th}}\,{\text{participant is in the high-dose}}\\ \qquad \quad{\text{fordadistrogene movaparvovec group}}\end{array}$$

$${Wi}={Pi}/(1-{Pi})\,{\mathrm{if}}\;{\mathrm{the}}\,{i}^{{\mathrm{th}}}\,{\mathrm{participant}}\;{\mathrm{is}}\;{\mathrm{in}}\;{\mathrm{the}}\;{\mathrm{external}}\;{\mathrm{control}}\;{\mathrm{cohort}}$$

where *Pi* is the probability of receiving fordadistrogene movaparvovec, conditional on the observed covariates. This creates a pseudo-control group that represents a random sample without any confounders. With these weights, fordadistrogene movaparvovec-treated participants received a weight of 1, and the external control participants received a weight of the odds of receiving fordadistrogene movaparvovec treatment. Thus, the population of fordadistrogene movaparvovec-treated participants serves as the reference population against which each of the fordadistrogene movaparvovec-treated and external control populations were standardized. If a participant in the external control cohort had a very high PS, a very large weight could be created. To reduce the impact of extreme weights, all weights with a value above the 95th percentile in the external control cohort were set equal to the 95th percentile. After trimming, the weights in the external control cohort were restandardized by dividing each individual weight by the sum of the total of weights in the external control cohort. After the PS weights were calculated, a linear model with a factor for treatment group was constructed to allow formal statistical comparisons between the fordadistrogene movaparvovec-treated and external control groups. In addition, doubly robust augmented inverse propensity weighting (AIPW) was performed^6^. The AIPW weights were included in the outcome regression model, using the treatment indicator and the same confounders (that is, the baseline covariates included in the PS model) to account for a doubly robust estimation.

The overlap of the distribution of the PS between fordadistrogene movaparvovec-treated and external control participants was visually inspected. For the PS weighting (IPTW-ATT) and doubly robust (AIPW) methods, the mean change from baseline for each treatment group along with the bootstrap s.e. and the bootstrap-bias-corrected 95% CI were provided. Additionally, the mean treatment group difference, associated bootstrap s.e., bootstrap-bias-corrected 95% CI and *P* value were provided.

A supplementary analysis plan for the external control analyses was created and finalized before the 1-year database lock.

### Statistics and reproducibility

The sample size of this ongoing, nonrandomized, open-label, ascending-dose, phase 1b study was based on clinical (rather than statistical) considerations to provide adequate safety, tolerability and pharmacodynamic data. No statistical method was used to predetermine sample size. No data were excluded from the analyses. The experiments were not randomized. The investigators were not blinded to allocation during the experiments and outcome assessment. Given that this is a phase 1b, open-label, single-arm study with a small sample size, reproducibility may be constrained in the traditional sense because of the lack of a control group and the small sample size. However, standard clinical trial protocols and rigorous data collection were implemented, including prespecified endpoints to ensure the integrity and reliability of the results. In addition, an external control analysis was conducted, which allowed comparison of the findings with data from similar populations. All methods and data are fully documented, enabling future replication and validation of the findings. There was no formal hypothesis testing in this study according to the original design of this first-in-human study. Nominal *P* values are reported. The 95% CIs for changes from baseline in secondary (dystrophin expression and distribution) and exploratory functional endpoints were calculated and interpreted as descriptive statistics.

The primary analyses of safety outcomes included all enrolled participants who received fordadistrogene movaparvovec. Analyses of dystrophin expression included all participants who received fordadistrogene movaparvovec and had baseline and at least one post-dose dystrophin parameter of interest reported. Descriptive statistics were calculated for the incidence of AEs, laboratory values and changes in dystrophin expression levels according to the fordadistrogene movaparvovec dose level. All analyses used observed data without imputation for missing data unless specified otherwise. Assay results below the lower limit of quantification were imputed as 0.5× the lower limit of quantification, assuming uniform distribution, for summary statistics and graphical presentation. The missing steroid regimen starting date in the external control cohort was imputed as 6 months before the screening day per the eligibility criteria of the previous interventional trials.

To contextualize treatment effects observed with high-dose fordadistrogene movaparvovec on the exploratory functional endpoints, an external control cohort was derived from 156 placebo-treated participants with DMD from two previous interventional trials^14,15^ The external control cohort used for the analysis of the 1-year exploratory efficacy endpoints was compiled from two previous DMD studies^15,32^, which were chosen based on their recency, reflection of the current standard of care, randomized controlled design, data provenance and data quality. An internal independent team was created to validate the external control cohort in a non-biased manner to ensure the interpretability of the results and the validity of the conclusions. This team included a clinician and a statistician within Pfizer who were not part of the NCT03362502 (that is, the current) study team, were not directly involved with the day-to-day activities of the study team and did not have access to post-baseline outcome data.

To minimize selection bias, the NCT03362502 eligibility criteria, including age, ambulatory status, glucocorticoid use, the ability to rise from the floor within 7 s and cardiac function (data permitting), along with a requirement for nonmissing NSAA scores at baseline and at 12 months, were applied to create a subset of participants for the external control cohort.

PS adjustment methods were used to balance measured characteristics between ambulatory high-dose fordadistrogene movaparvovec participants and the external control cohort. The average causal treatment effect for high-dose-fordadistrogene movaparvovec-treated participants was estimated using IPTW-ATT. Prespecified covariates (that is, screening age, baseline NSAA total score, rise from floor velocity and 10-m run/walk velocity) were included in the PS model. Candidate covariates were selected based on their potential to influence treatment group selection or to be related to outcome risk. To address the potential over-influence of large weights, all weights above the 95th percentile in the external control cohort were truncated to the 95th percentile and then normalized according to the total of the weights in the group. Functional endpoints were analyzed using a linear regression model with treatment indicator and IPTW-ATT weights to adjust for differences between the high-dose fordadistrogene movaparvovec and external control groups. To evaluate the robustness of the findings, sensitivity analyses were conducted using doubly robust estimation to account for covariates in the outcome models. Bootstrap methods were used to obtain the estimated s.e. for the treatment effect and 95% CI without making underlying distributional assumptions. Balance of baseline variables between fordadistrogene movaparvovec-treated and external control participants in the unweighted and PS-weighted samples was examined using the standardized mean difference to quantify the magnitude of the difference between the two groups. The detailed methods used to derive and analyze the external control cohort can be found in the Methods.

Data as of 30 September 2022 were analyzed using SAS v.9.4 (SAS Institute) or R v. 4.1.3 (R Foundation)^39^.

### Reporting summary

Further information on research design is available in the Nature Portfolio Reporting Summary linked to this article.

## Online content

Any methods, additional references, Nature Portfolio reporting summaries, source data, extended data, supplementary information, acknowledgements, peer review information; details of author contributions and competing interests; and statements of data and code availability are available at 10.1038/s41591-025-03750-3.

## Supplementary information

Supplementary Information Supplementary Tables 1–5 and Figs. 1–3.

Reporting Summary

## Figures and Tables

**Extended Data Table 1:**
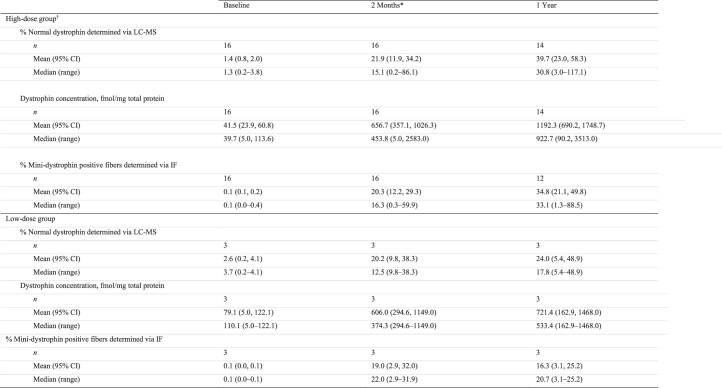
Analyses of dystrophin expression in ambulatory study participants in the current phase 1b study

*One biopsy sample was collected at 3 months (allowed per protocol).

^†^Two data points were missing at the 1-year primary analysis because samples were deemed unacceptable for fiber detection by the independent vendor (one had poor fiber morphology and one was a small muscle section with low number of mostly longitudinal fragmented fibers). Since positive fiber data were not available for these samples, the corresponding LC–MS results were removed from the primary analyses (per the statistical analysis plan) before examination of the results.

CI, confidence interval; IF, immunofluorescence; LC–MS, liquid chromatography–mass spectrometry.

**Extended Data Table 2:**
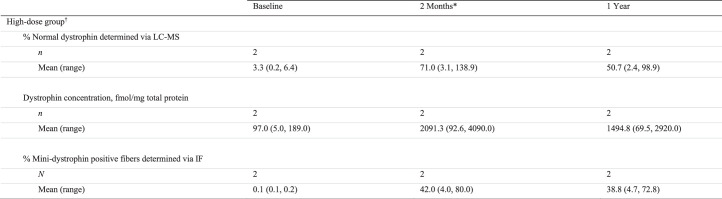
Analyses of dystrophin expression in nonambulatory study participants in the current phase 1b study

IF, immunofluorescence; LC-MS, liquid chromatography–mass spectrometry.

**Extended Data Table 3:**
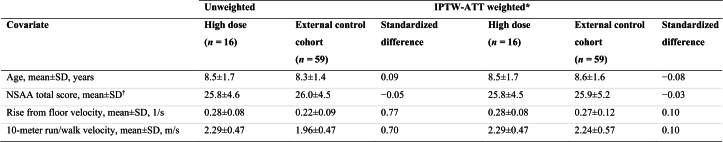
Baseline covariate balance before and after IPTW-ATT adjustments for ambulatory high-dose participants versus external control cohort

A standardized mean difference of <0.1 represents acceptable balance between groups.

*ATT weight above the 95th percentile in the external control cohort was truncated to the 95th percentile and then normalized by the total of weights in the group.

^†^NSAA total score is based on assessment of 17 ambulatory actions and ranges from 0 to 34, with higher score indicating greater functional ability.

IPTW-ATT, inverse probability treatment weighting-average treatment effect in the treated; NSAA, North Star Ambulatory Assessment; SD, standard deviation.

**Extended Data Table 4:**
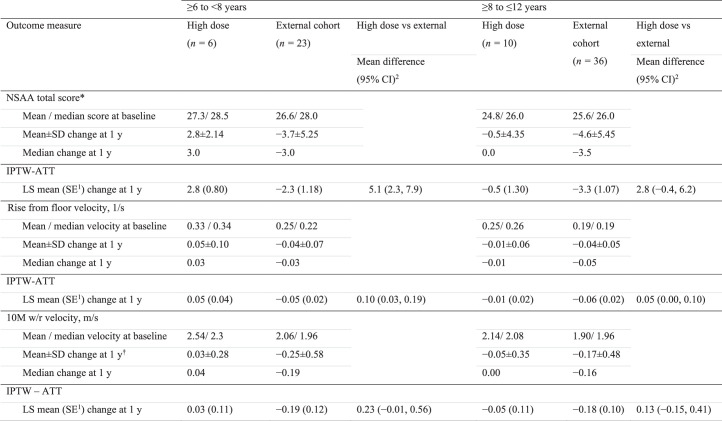
Change (from baseline to 1 year) in functional assessments in ambulatory high-dose participants and in the external control cohort

Stratified by baseline age group.

^1^Bootstrap standard error; ^2^Bootstrap bias corrected 95% confidence interval.

IPTW, inverse probability of treatment weighting. IPTW-ATT, inverse probability treatment weighting-average treatment effect in the treated; CI, confidence interval; LS mean, least square mean; NSAA, North Star Ambulatory Assessment; SE, standard error.

## Data Availability

Upon reasonable request and subject to review, Pfizer will provide the data that support the findings of this study. Subject to certain criteria, conditions and exceptions, Pfizer may also provide access to the related individual deidentified participant data from Pfizer-sponsored global interventional clinical studies conducted for (1) medicines, vaccines and medical devices for indications that have been approved in the United States or European Union or (2) in programs that have been terminated (that is, development for all indications has been discontinued). Pfizer will also consider requests for the protocol, data dictionary and statistical analysis plan (see https://www.pfizer.com/science/clinical-trials/trial-data-and-results for more information). Data from Pfizer trials may be requested 24 months after study completion. Deidentified participant data will be made available to researchers whose proposals meet the research criteria and other conditions, and for which an exception does not apply, via a secure portal. To gain access, data requestors must enter into a data access agreement with Pfizer.
