# Supplementary material for: AAV mini-dystrophin gene therapy for Duchenne muscular dystrophy: a phase 1b trial
Source: Nat Med. 2025 Jun 27;31(8):2712–21. doi: 10.1038/s41591-025-03750-3 (PMC12353823; doi:10.1038/s41591-025-03750-3)
Supplement: Supplementary file 1 — Supplementary Tables 1–5 and Figs. 1–3. [file 41591_2025_3750_MOESM1_ESM.pdf]

nature medicine

Supplementary information https://doi.org/10.1038/s41591-025-03750-3

# AAV mini-dystrophin gene therapy for Duchenne muscular dystrophy: a phase 1b trial

In the format provided by the authors and unedited

# SUPPLEMENTARY MATERIAL

## SUPPLEMENTARY TABLES

### Table 1. *DMD* mutations identified in individual participants

| Cohort | Mutation* | Mutation category |
|---|---|---|
| Low dose ambulatory | c.4538_4541delGTGA, p.Ser1513Lysfs*2 | Indel |
| Low dose ambulatory | c.5917C>T, p.Gln1973* | Nonsense |
| Low dose ambulatory | deletion exons 46–47 | Deletion (out of frame) |
| High dose ambulatory | deletion exons 49–52 | Deletion (out of frame) |
| High dose ambulatory | deletion exons 50–52 | Deletion (out of frame) |
| High dose ambulatory | deletion exons 48–52 | Deletion (out of frame) |
| High dose ambulatory | deletion exons 52–54 | Deletion (out of frame) |
| High dose ambulatory | c.5448+67A>G | Cryptic splice donor with pseudoexon insertion[6] |
| High dose ambulatory | c.1259dup, p.Gln421Thrfs*18 | Indel |
| High dose ambulatory | deletion exons 46–52 | Deletion (out of frame) |
| High dose ambulatory | deletion exons 14–17 | Deletion (out of frame) |
| High dose ambulatory | duplication exons 8–9 | Duplication (out of frame) |
| High dose ambulatory | duplication exon 18 | Duplication (out of frame) |
| High dose ambulatory | duplication exons 8–9 | Duplication (out of frame) |
| High dose ambulatory | c.1865C>G, p.Ser622* | Nonsense |
| High dose ambulatory | c.1865C>G, p.Ser622* | Nonsense |
| High dose ambulatory | deletion exons 48–52 | Deletion (out of frame) |
| High dose ambulatory | c.5758C>T, p.Gln1920* | Nonsense |
| High dose ambulatory | c.5758C>T, p.Gln1920* | Nonsense |
| High dose non-ambulatory | c.5697dup, p.Leu1900Ilefs*6 | Indel |

| | | |
|---|---|---|
| High dose non-ambulatory | c.6911del, Lys2304Argfs*17 | Indel |
| High dose non-ambulatory | deletion exons 46–47 | Deletion (out of frame) |

Variant positions in the Mutation column refer to RefSeq NM_004006.2

**Table 2. Incidence of laboratory test abnormalities**

| | Ambulatory participants (*N*= *19*) | | | | | | Non-ambulatory participants (*N*= *3*) | |
|---|---|---|---|---|---|---|---|---|
| | Low dose (*n* = 3) | | High dose (*n* = 16) | | All (*N* = 19) | | High dose (*N* = *3*) | |
| Laboratory abnormality | N | n (%) | N | n (%) | N | n (%) | N | n (%) |
| Hemoglobin (g/dL) <0.8xLLN | 3 | 0 | 16 | 1 (6.3) | 19 | 1 (5.3) | 3 | 1 (33.3) |
| Hematocrit (%) <0.8xLLN | 3 | 0 | 16 | 2 (12.5) | 19 | 2 (10.5) | 3 | 1 (33.3) |
| Erythrocytes (10^6/mm3) <0.8xLLN | 3 | 0 | 16 | 1 (6.3) | 19 | 1 (5.3) | 3 | 1 (33.3) |
| Platelets (10^3/mm3) <0.5x LLN | 3 | 0 | 16 | 5 (31.3) | 19 | 5 (26.3) | 3 | 1 (33.3) |
| Erythrocyte Cell Morphology (No Unit) >0 | 2 | 0 | 14 | 1 (7.1) | 16 | 1 (6.3) | 3 | 1 (33.3) |
| Leukocytes (10^3/mm3) >1.5x ULN | 3 | 0 | 16 | 2 (12.5) | 19 | 2 (10.5) | 3 | 1 (33.3) |
| Lymphocytes (10^3/mm3) <0.8x LLN | 3 | 1 (33.3) | 16 | 6 (37.5) | 19 | 7 (36.8) | 3 | 2 (66.7) |
| Lymphocytes (10^3/mm3) >1.2x ULN | 3 | 0 | 16 | 2 (12.5) | 19 | 2 (10.5) | 3 | 1 (33.3) |
| Lymphocytes Atypical (10^3/mm3) >0 | 1 | 1 (100.0) | 9 | 9 (100.0) | 10 | 10 (100.0) | 2 | 2 (100.0) |
| Neutrophils (10^3/mm3) <0.8x LLN | 3 | 0 | 16 | 2 (12.5) | 19 | 2 (10.5) | 3 | 0 |
| Neutrophils (10^3/mm3) >1.2x ULN | 3 | 1 (33.3) | 16 | 7 (43.8) | 19 | 8 (42.1) | 3 | 2 (66.7) |
| Neutrophils band form (10^3/mm$^3$) >0.27 | | | | | | | 1 | 1 (100.0) |
| Basophils (10^3/mm3) >1.2x ULN | 3 | 0 | 16 | 1 (6.3) | 19 | 1 (5.3) | 3 | 0 |
| Eosinophils (10^3/mm3) >1.2x ULN | 3 | 1 (33.3) | 16 | 2 (12.5) | 19 | 3 (15.8) | 3 | 0 |
| Monocytes (10^3/mm3) >1.2x ULN | 3 | 0 | 16 | 12 (75.0) | 19 | 12 (63.2) | 3 | 3 (100.0) |
| Activated Partial Thromboplastin Time (sec) >1.1x ULN | 3 | 1 (33.3) | 16 | 1 (6.3) | 19 | 2 (10.5) | 3 | 0 |
| Prothrombin Time (sec) >1.1x ULN | 3 | 1 (33.3) | 16 | 7 (43.8) | 19 | 8 (42.1) | 3 | 2 (66.7) |
| Neutrophils -TOTAL COUNT (10^3/mm3) <1.35 | 3 | 0 | 16 | 2 (12.5) | 19 | 2 (10.5) | 3 | 0 |
| Neutrophils -TOTAL COUNT (10^3/mm3) >8.15 | 3 | 2 (66.7) | 16 | 14 (87.5) | 19 | 16 (84.2) | 3 | 2 (66.7) |
| Myelocytes (10^3/mm3) >0 | 0 | 0 | 1 | 1 (100.0) | 1 | 1 (100.0) | 0 | 0 |
| Haptoglobin (mg/dL) <30 | 0 | 0 | 13 | 3 (23.1) | 13 | 3 (23.1) | 3 | 1 (33.3) |
| Haptoglobin (mg/dL) >200 | 0 | 0 | 13 | 10 (76.9) | 13 | 10 (76.9) | 3 | 1 (33.3) |

| | | | | | | | | |
|---|---|---|---|---|---|---|---|---|
| Bilirubin (mg/dL) >1.5x ULN | | | | | | | 3 | 1 (33.3) |
| Direct bilirubin (mg/dL) | | | | | | | 2 | 1 (50.0) |
| Aspartate Aminotransferase (U/L) >3.0x ULN | 3 | 3 (100.0) | 16 | 16 (100.0) | 19 | 19 (100.0) | 3 | 3 (100.0) |
| Alanine Aminotransferase (U/L) >3.0x ULN | 3 | 3 (100.0) | 16 | 16 (100.0) | 19 | 19 (100.0) | 3 | 3 (100.0) |
| Gamma Glutamyl Transferase (U/L) >3.0x ULN | 3 | 0 | 16 | 1 (6.3) | 19 | 1 (5.3) | 3 | 1 (33.3) |
| Protein (g/dL) <0.8x LLN | 3 | 0 | 16 | 2 (12.5) | 19 | 2 (10.5) | 3 | 0 |
| Albumin (g/dL) | | | | | | | 3 | 1 (33.3) |
| Urea Nitrogen (mg/dL) >1.3x ULN | 3 | 0 | 16 | 1 (6.3) | 19 | 1 (5.3) | 3 | 1 (33.3) |
| Creatinine (mg/dL) >1.3x ULN | 3 | 0 | 16 | 1 (6.3) | 19 | 1 (5.3) | 3 | 1 (33.3) |
| Urate (mg/dL) >1.2x ULN | 3 | 0 | 16 | 6 (37.5) | 19 | 6 (31.6) | 3 | 0 |
| Sodium (mEq/L) <0.95x LLN | 3 | 0 | 16 | 1 (6.3) | 19 | 1 (5.3) | 3 | 2 (66.7) |
| Potassium (mEq/L) <0.9x LLN | 3 | 0 | 16 | 1 (6.3) | 19 | 1 (5.3) | 3 | 1 (33.3) |
| Calcium (mg/dL) <0.9x LLN | | | | | | | 3 | 1 (33.3) |
| Phosphate (mg/dL) <0.8x LLN | 3 | 0 | 16 | 2 (12.5) | 19 | 2 (10.5) | 3 | 1 (33.3) |
| Bicarbonate (mEq/L) <0.9x LLN | 3 | 0 | 16 | 2 (12.5) | 19 | 2 (10.5) | 3 | 2 (66.7) |
| Bicarbonate (mEq/L) >1.1x ULN | 3 | 0 | 16 | 1 (6.3) | 19 | 1 (5.3) | 3 | 0 |
| Glucose (mg/dL) >1.5x ULN | 3 | 0 | 16 | 4 (25.0) | 19 | 4 (21.1) | 3 | 2 (66.7) |
| Creatine Kinase (U/L) >2.0x ULN | 3 | 3 (100.0) | 16 | 16 (100.0) | 19 | 19 (100.0) | 3 | 3 (100.0) |
| Amylase (U/L) | | | | | | | 3 | 3 (100.0) |
| Triacylglycerol Lipase (U/L) >1.5x ULN | | | | | | | 3 | 1 (33.3) |
| C-Reactive Protein High Sens (mg/dL) >1.1x ULN | 3 | 0 | 16 | 3 (18.8) | 19 | 3 (15.8) | 3 | 2 (66.7) |
| Cystatin C (mg/L) >1.2x ULN | 3 | 0 | 16 | 3 (18.8) | 19 | 3 (15.8) | 3 | 1 (33.3) |
| Creatine Kinase MB (ug/L) >2.0x ULN | 3 | 3 (100.0) | 16 | 16 (100.0) | 19 | 19 (100.0) | 2 | 2 (100.0) |
| Glutamate Dehydrogenase (U/L) >1.0x ULN | 3 | 2 (66.7) | 16 | 10 (62.5) | 19 | 12 (63.2) | 3 | 3 (100.0) |
| Glutamate Dehydrogenase (U/L) >2.0x ULN | 3 | 0 | 16 | 5 (31.3) | 19 | 5 (26.3) | 3 | 2 (66.7) |
| Glutamate Dehydrogenase (U/L) >3.0x ULN | 3 | 0 | 16 | 3 (18.8) | 19 | 3 (15.8) | 3 | 2 (66.7) |
| Ferritin (ng/mL) <23.9 | 0 | 0 | 13 | 8 (61.5) | 13 | 8 (61.5) | 3 | 0 |
| Ferritin (ng/mL) >336.2 | 0 | 0 | 13 | 11 (84.6) | 13 | 11 (84.6) | 3 | 3 (100.0) |
| URINE Ketones (No Unit) ≥1 | 3 | 0 | 16 | 7 (43.8) | 19 | 7 (36.8) | 3 | 3 (100.0) |

| | | | | | | | | |
|---|---|---|---|---|---|---|---|---|
| URINE Protein (Scalar) ≥1 | 3 | 0 | 16 | 2 (12.5) | 19 | 2 (10.5) | 3 | 1 (33.3) |
| URINE Hemoglobin (No Unit) ≥1 | 3 | 0 | 16 | 4 (25.0) | 19 | 4 (21.1) | 3 | 1 (33.3) |
| URINE Nitrite (No Unit) ≥1 | 3 | 0 | 16 | 1 (6.3) | 19 | 1 (5.3) | 3 | 0 |
| URINE Erythrocytes (/HPF) ≥20 | 1 | 0 | 6 | 1 (16.7) | 7 | 1 (14.3) | 3 | 0 |
| URINE Leukocytes (/HPF) ≥20 | 0 | 0 | 12 | 1 (8.3) | 12 | 1 (8.3) | 2 | 0 |
| Granular Casts (/LPF) >1 | 0 | 0 | 1 | 1 (100.0) | 1 | 1 (100.0) | 0 | 0 |
| Hyaline Casts (/LPF) >1 | 0 | 0 | 2 | 1 (50.0) | 2 | 1 (50.0) | 0 | 0 |
| Calcium Oxalate Crystals (/LPF) ≥0.5 | 2 | 2 (100.0) | 2 | 2 (100.0) | 4 | 4 (100.0) | 1 | 1 (100.0) |
| Amorphous Crystals (/LPF) ≥0.5 | 0 | 0 | 7 | 7 (100.0) | 7 | 7 (100.0) | 1 | 1 (100.0) |
| Urine Microscopic Exam (Scalar) ≥1 | 2 | 2 (100.0) | 14 | 14 (100.0) | 16 | 16 (100.0) | 3 | 3 (100.0) |

N = total number of participants with at least one observation of the given laboratory test while on study treatment or during lag time. n = number of participants with a laboratory abnormality meeting specified criteria while on study treatment or during lag time. Percentages were displayed for the laboratory tests having a category with greater or equal to 1 evaluable participant.

HPF, high power field; LLN, lower limit of normal; LPF, low power field; ULN, upper limit of normal.

**Table 3. Post-baseline vital signs**

| | Ambulatory participants (*N = 19)* | | | | | | Non-ambulatory participants (*N* = *3*) | |
|---|---|---|---|---|---|---|---|---|
| | Low dose (*n* = 3) | | High dose (*n* = 16) | | All (*N* = 19) | | High dose (*N* = *3*) | |
| Parameter | N | n (%) | N | n (%) | N | n (%) | N | n (%) |
| Respiratory rate (breaths/minute) | | | | | | | | |
| Value <14 breaths/min for aged 6 to 12 years | 3 | 0 | 16 | 0 | 19 | 0 | 3 | 0 |
| Value >22 breaths/min for aged 6 to 12 years | 3 | 3 (100.0) | 16 | 16 (100.0) | 19 | 19 (100.0) | 3 | 0 |
| Value <12 breaths/min for aged 13 to 18 years | 3 | 0 | 16 | 0 | 19 | 0 | 3 | 0 |
| Value >20 breaths/min for aged 13 to 18 years | 3 | 0 | 16 | 0 | 19 | 0 | 3 | 3 (100.0) |
| Supine diastolic BP (mmHg) | | | | | | | | |
| Value <50 mmHg | 3 | 1 (33.3) | 16 | 5 (31.3) | 19 | 6 (31.6) | 2 | 0 |
| Change ≥20 mmHg increase | 3 | 0 | 16 | 2 (12.5) | 19 | 2 (10.5) | 2 | 0 |
| Change ≥20 mmHg decrease | 3 | 1 (33.3) | 16 | 2 (12.5) | 19 | 3 (15.8) | 2 | 1 (50.0) |
| Supine HR (BPM) | | | | | | | | |
| Value <40 bpm | 3 | 0 | 16 | 0 | 19 | 0 | 2 | 0 |
| Value >120 bpm | 3 | 0 | 16 | 1 (6.3) | 19 | 1 (5.3) | 2 | 1 (50.0) |
| Supine systolic BP (mmHg) | | | | | | | | |
| Value <70 mmHg + 2*Age for aged 2 to 10 years | 3 | 0 | 16 | 0 | 19 | 0 | 2 | 0 |
| Value <90 mmHg for aged 11 years and older | 3 | 0 | 16 | 1 (6.3) | 19 | 1 (5.3) | 2 | 0 |
| Change ≥30 mmHg increase from baseline | 3 | 0 | 16 | 5 (31.3) | 19 | 5 (26.3) | 2 | 0 |
| Change ≥30 mmHg decrease from baseline | 3 | 1 (33.3) | 16 | 0 | 19 | 1 (5.3) | 2 | 0 |

Baseline was defined as the last pre-dose measurement.

BP, blood pressure; BPM, beats per minute; HR, heart rate

**Table 4. Summary of ECG data**

| | Ambulatory participants (*N*= *19*) | | | | | | Non-ambulatory participants (*N*= *3*) | |
|---|---|---|---|---|---|---|---|---|
| | Low dose (*n* = 3) | | High dose (*n* = 16) | | All (*N* = 19) | | High dose (*N* = *3*) | |
| Parameter | N | n (%) | N | n (%) | N | n (%) | N | n (%) |
| PR interval, aggregate (msec) | | | | | | | | |
| Value ≥300 | 3 | 0 | 16 | 0 | 19 | 0 | 3 | 0 |
| Baseline > 200 and % Change ≥25% | 3 | 0 | 16 | 0 | 19 | 0 | 3 | 0 |
| Baseline ≤200 and % Change ≥50% | 3 | 0 | 16 | 0 | 19 | 0 | 3 | 0 |
| QRS duration, aggregate (msec) | | | | | | | | |
| Value ≥140 | 3 | 0 | 16 | 0 | 19 | 0 | 3 | 0 |
| % Change ≥50% | 3 | 0 | 16 | 0 | 19 | 0 | 3 | 0 |
| QTCF interval, aggregate (msec) | | | | | | | | |
| 450≤Value < 480 | 3 | 0 | 16 | 0 | 19 | 0 | 3 | 0 |
| 480 ≤Value < 500 | 3 | 0 | 16 | 0 | 19 | 0 | 3 | 0 |
| Value ≥500 | 3 | 0 | 16 | 0 | 19 | 0 | 3 | 0 |
| 30 ≤Change < 60 | 3 | 1 (33.3) | 16 | 2 (12.5) | 19 | 3 (15.8) | 3 | 0 |
| Change ≥60 | 3 | 0 | 16 | 0 | 19 | 0 | 3 | 0 |

Baseline was defined as the average of the last triplicate ECG measurements collected prior to dose.

**Table 5. Antibodies, controls and concentrations used for mini-DYS/laminin dual staining panel in the immunofluorescence assay**

| Primary Antibody | | | | Secondary Antibody | | | |
|---|---|---|---|---|---|---|---|
| **Antibody Description (Host/antigen/isotype/clone)** | **Source** | Catalog # | Working Concentration | Description (Host/isotype/fluorophore/clone) | Source | Catalog # | Working Concentration |
| Mouse anti-Mini-DYS, Mouse IgG2b, Clone A.01 25H5.H6 | Pfizer | --- | 10 ug/ml | Goat anti-mouse Alexa Fluor 594 | Thermofisher | A11032 | 5 ug/ml |
| Anti-Laminin 2 alpha, Rat IgG1, clone: 4H8-2 | Abcam | ab11576 | 5.5 ug/ml | Rabbit anti-rat Alexa Fluor 488 | Thermofisher | A21210 | 5 ug/ml |

## SUPPLEMENTARY FIGURES

**Figure 1. Fordadistrogene movaparvovec vector structure and sequence.**

Fordadistrogene movaparvovec transgene sequence of 4899bps includes the mini-dystrophin coding sequence (green highlight) of 3978bps.

| | | | | |
|---|---|---|---|---|
| *AAV2 ITR* | **Hybrid CK promoter** | **Minidystrophin coding sequence** | Polyadenylation sequence | *AAV2 ITR* |

*GGGGGGGGGG GGGGGGGTTG GCCACTCCCT CTCTGCGCGC TCGCTCGCTC*

*ACTGAGGCCG GGCGACCAAA GGTCGCCCGA CGCCCGGGCT TTGCCCGGGC*

*GGCCTCAGTG AGCGAGCGAG CGCGCAGAGA GGGAGTGGCC AACTCCATCA*

*CTAGGGGTTC CT*CAGATCTG AATTCGGTAC C**CCACTACGG GTCTAGGCTG**

**CCCATGTAAG GAGGCAAGGC CTGGGGACAC CCGAGATGCC TGGTTATAAT**

**TAACCCAGAC ATGTGGCTGC CCCCCCCCCC CCCAACACCT GCTGCCTCTA**

**AAAATAACCC TGTCCCTGGT GGATCCCCTG CATGCGAAGA TCTTCGAACA**

**AGGCTGTGGG GGACTGAGGG CAGGCTGTAA CAGGCTTGGG GGCCAGGGCT**

**TATACGTGCC TGGGACTCCC AAAGTATTAC TGTTCCATGT TCCCGGCGAA**

**GGGCCAGCTG TCCCCCGCCA GCTAGACTCA GCACTTAGTT TAGGAACCAG**

**TGAGCAAGTC AGCCCTTGGG GCAGCCCATA CAAGGCCATG GGGCTGGGCA**

**AGCTGCACGC CTGGGTCCGG GGTGGGCACG GTGCCCGGGC AACGAGCTGA**

**AAGCTCATCT GCTCTCAGGG GCCCCTCCCT GGGGACAGCC CCTCCTGGCT**

**AGTCACACCC TGTAGGCTCC TCTATATAAC CCAGGGGCAC AGGGGCTGCC**

**CTCATTCTAC CACCACCTCC ACAGCACAGA CAGACACTCA GGAGCCAGCC**

**AGCGTCGA**GC GGCCGATCCG CCACC**ATGCT TTGGTGGGAG GAAGTGGAGG**

**ACTGCTACGA GAGAGAGGAC GTGCAGAAGA AAACCTTCAC CAAGTGGGTG**

**AACGCCCAGT TCAGCAAGTT CGGCAAGCAG CACATCGAGA ACCTGTTCAG**

**CGACCTGCAG GATGGCAGGA GACTGCTGGA CCTGCTGGAG GGCCTGACCG**

**GCCAGAAGCT GCCCAAGGAG AAGGGCAGCA CCAGAGTGCA CGCCCTGAAC**

**AACGTGAACA AGGCCCTGAG AGTGCTGCAG AACAACAACG TGGACCTGGT**

**GAACATCGGC AGCACCGACA TCGTGGACGG CAACCACAAG CTGACCCTGG**

**GCCTGATCTG GAACATCATC CTGCACTGGC AGGTGAAGAA CGTGATGAAG**

**AACATCATGG CCGGCCTGCA GCAGACCAAC AGCGAGAAGA TCCTGCTGAG**

**CTGGGTGAGG CAGAGCACCA GAAACTACCC CCAGGTGAAC GTGATCAACT**

**TCACCACCTC CTGGAGCGAC GGCCTGGCCC TGAACGCCCT GATCCACAGC**

**CACAGACCCG ACCTGTTCGA CTGGAACAGC GTGGTGTGTC AGCAGAGCGC**

**CACCCAGAGA CTGGAGCACG CCTTCAACAT CGCCAGATAC CAGCTGGGCA**

**TCGAGAAGCT GCTGGACCCC GAGGACGTGG ACACCACCTA CCCCGACAAG**

**AAAAGCATCC TCATGTACAT TACCAGCCTG TTCCAGGTGC TGCCCCAGCA**

**GGTGTCCATC GAGGCCATCC AGGAAGTGGA AATGCTGCCC AGGCCCCCCA**

**AAGTGACCAA GGAGGAGCAC TTCCAGCTGC ACCACCAGAT GCACTACAGC**

**CAGCAGATCA CAGTGAGCCT GGCCCAGGGC TATGAGAGAA CCAGCAGCCC**

**CAAGCCCAGA TTCAAGAGCT ACGCCTACAC CCAGGCCGCC TACGTGACCA**

**CCTCCGACCC CACCAGAAGC CCCTTCCCCA GCCAGCACCT GGAGGCCCCC**

**GAGGACAAGA GCTTCGGCAG CAGCCTGATG GAGAGCGAAG TGAACCTGGA**

**CAGATACCAG ACCGCCCTGG AGGAAGTGCT GTCCTGGCTG CTGAGCGCCG**

AGGACACCCT GCAGGCCCAG GGCGAGATCA GCAACGACGT GGAAGTGGTG

AAGGACCAGT TCCACACCCA CGAGGGCTAC ATGATGGATC TGACCGCCCA

CCAGGGCAGA GTGGGCAATA TCCTGCAGCT GGGCAGCAAG CTGATCGGCA

CCGGCAAGCT GAGCGAGGAC GAGGAGACCG AAGTGCAGGA GCAGATGAAC

CTGCTGAACA GCAGATGGGA GTGCCTGAGA GTGGCCAGCA TGGAGAAGCA

GAGCAACCTG CACAGAGTGC TGATGGACCT GCAGAACCAG AAGCTGAAGG

AGCTGAACGA CTGGCTGACC AAGACCGAGG AGCGGACCAG AAAGATGGAG

GAGGAGCCCC TGGGCCCCGA CCTGGAGGAC CTGAAGAGAC AGGTGCAGCA

GCACAAAGTG CTGCAGGAGG ACCTGGAGCA GGAGCAGGTG CGCGTGAACA

GCCTGACCCA CATGGTGGTG GTCGTGGACG AGAGCAGCGG CGACCACGCC

ACAGCCGCCC TGGAAGAGCA GCTGAAAGTG CTGGGCGACA GATGGGCCAA

TATTTGTAGG TGGACCGAGG ACAGATGGGT GCTGCTGCAG GACCAGCCCG

ACCTGGCCCC TGGCCTGACC ACCATCGGCG CCAGCCCCAC CCAGACCGTG

ACCCTGGTGA CCCAGCCCGT GGTGACAAAG GAGACCGCCA TCAGCAAGCT

GGAGATGCCC AGCTCCCTGA TGCTGGAAGT GCCCACCCAC CGCCTGCTCC

AGCAGTTCCC CCTGGACCTG GAGAAGTTCC TGGCCTGGCT GACCGAGGCC

GAAACCACCG CCAATGTGCT CCAGGACGCC ACTAGAAAGG AGAGGCTGCT

GGAGGACAGC AAGGGCGTGA AAGAGCTGAT GAAGCAGTGG CAGGATCTGC

AGGGCGAAAT CGAGGCCCAC ACCGACGTGT ACCACAACCT GGACGAGAAC

AGCCAGAAGA TTCTGAGGAG CCTGGAGGGC AGCGACGACG CCGTCCTGCT

CCAGAGGAGG CTGGACAACA TGAACTTCAA GTGGAGCGAG CTGCGGAAGA

AGAGCCTGAA CATCCGGAGC CACCTGGAAG CCAGCAGCGA CCAGTGGAAG

AGACTGCACC TGAGCCTGCA GGAGCTGCTG GTGTGGCTGC AGCTGAAGGA

CGACGAGCTG AGCAGACAGG CCCCCATCGG CGGCGACTTC CCCGCCGTGC

AGAAGCAGAA CGACGTGCAC CGGGCCTTCA AGAGGGAGCT GAAAACCAAG
GAACCCGTGA TCATGAGCAC CCTGGAGACA GTGCGGATCT TCCTGACCGA
GCAGCCCCTG GAGGGACTGG AGAAGCTGTA CCAGGAGCCC AGAGAGCTGC
CCCCCGAGGA GAGAGCCCAG AACGTGACCA GGCTGCTGAG AAAGCAGGCC
GAGGAAGTGA ATACCGAGTG GGAGAAGCTG AATCTGCACA GCGCCGACTG
GCAGAGAAAG ATCGACGAGA CCCTGGAGAG ACTCCAGGAA CTGCAGGAAG
CCACCGACGA GCTGGACCTG AAGCTGAGAC AGGCCGAAGT GATCAAGGGC
AGCTGGCAGC CTGTGGGCGA TCTGCTGATC GACTCCCTGC AGGATCACCT
GGAGAAAGTG AAGGCCCTGC GGGGCGAGAT CGCCCCCCTG AAGGAGAATG
TGAGCCACGT GAACGACCTG GCCAGACAGC TGACCACCCT GGGCATCCAG
CTGAGCCCCT ACAACCTGAG CACACTGGAG GATCTGAACA CCCGGTGGAA
ACTGCTGCAG GTGGCCGTGG AGGATAGAGT GAGGCAGCTG CACGAAGCCC
ACAGAGACTT CGGCCCTGCC TCCCAGCACT TCCTGAGCAC CAGCGTGCAG
GGCCCCTGGG AGAGAGCCAT CTCCCCCAAC AAAGTGCCCT ACTACATCAA
CCACGAGACC CAGACCACCT GCTGGGACCA CCCTAAGATG ACCGAGCTGT
ATCAGAGCCT GGCCGACCTG AACAATGTGC GGTTCAGCGC CTACAGAACC
GCCATGAAGC TGCGGAGACT GCAGAAGGCC CTGTGCCTGG ATCTGCTGAG
CCTGAGCGCC GCCTGCGACG CCCTGGACCA GCACAACCTG AAGCAGAATG
ACCAGCCCAT GGACATCCTG CAGATCATCA ACTGCCTGAC CACAATCTAC
GACCGGCTGG AACAGGAGCA CAACAACCTG GTGAATGTGC CCCTGTGCGT
GGACATGTGC CTGAATTGGC TGCTGAACGT GTACGACACC GGCAGGACCG
GCAGAATCCG CGTGCTGAGC TTCAAGACCG GCATCATCAG CCTGTGCAAG
GCCCACCTGG AGGATAAGTA CCGCTACCTG TTCAAGCAGG TGGCCAGCAG
CACCGGCTTC TGCGATCAGA GGAGACTGGG CCTGCTGCTG CACGATAGCA

**TCCAGATCCC TAGGCAGCTG GGCGAAGTGG CCAGCTTTGG CGGCAGCAAC**

**ATCGAGCCCT CTGTGAGGAG CTGCTTCCAG TTCGCCAACA ACAAGCCCGA**

**GATCGAGGCC GCCCTGTTCC TGGACTGGAT GAGGCTGGAG CCTCAGAGCA**

**TGGTGTGGCT GCCTGTGCTG CACAGAGTGG CCGCCGCCGA GACCGCCAAG**

**CACCAGGCCA AGTGCAATAT CTGCAAGGAG TGCCCCATCA TCGGCTTCCG**

**GTACAGGAGC CTGAAGCACT TCAACTACGA CATCTGCCAG AGCTGCTTTT**

**TCAGCGGCAG AGTGGCCAAG GGCCACAAAA TGCACTACCC CATGGTGGAG**

**TACTGCACCC CCACCACCTC CGGCGAGGAT GTGAGAGACT TCGCCAAAGT**

**GCTGAAGAAT AAGTTCCGGA CCAAGCGGTA CTTTGCCAAG CACCCCAGGA**

**TGGGCTACCT GCCCGTGCAG ACCGTGCTGG AAGGCGACAA CATGGAGACC**

**TGA**TGAGGAG CTCGAGAGGC CTAATAAAGA GCTCAGATGC ATCGATCAGA

GTGTGTTGGT TTTTTGTGTG AGATCTG*AGG AACCCCTAGT GATGGAGTTG*

*GCCACTCCCT CTCTGCGCGC TCGCTCGCTC ACTGAGGCCG CCCGGGCAAA*

*GCCCGGGCGT CGGGCGACCT TTGGTCGCCC GGCCTCAGTG AGCGAGCGAG*

*CGCGCAGAGA GGGAGTGGCC AACCCCCCCC CCCCCCCC*

**Figure 2. Representative immunofluorescence images from an ambulatory participant showing expression of mini-dystrophin protein after treatment with fordadistrogene movaparvovec.**

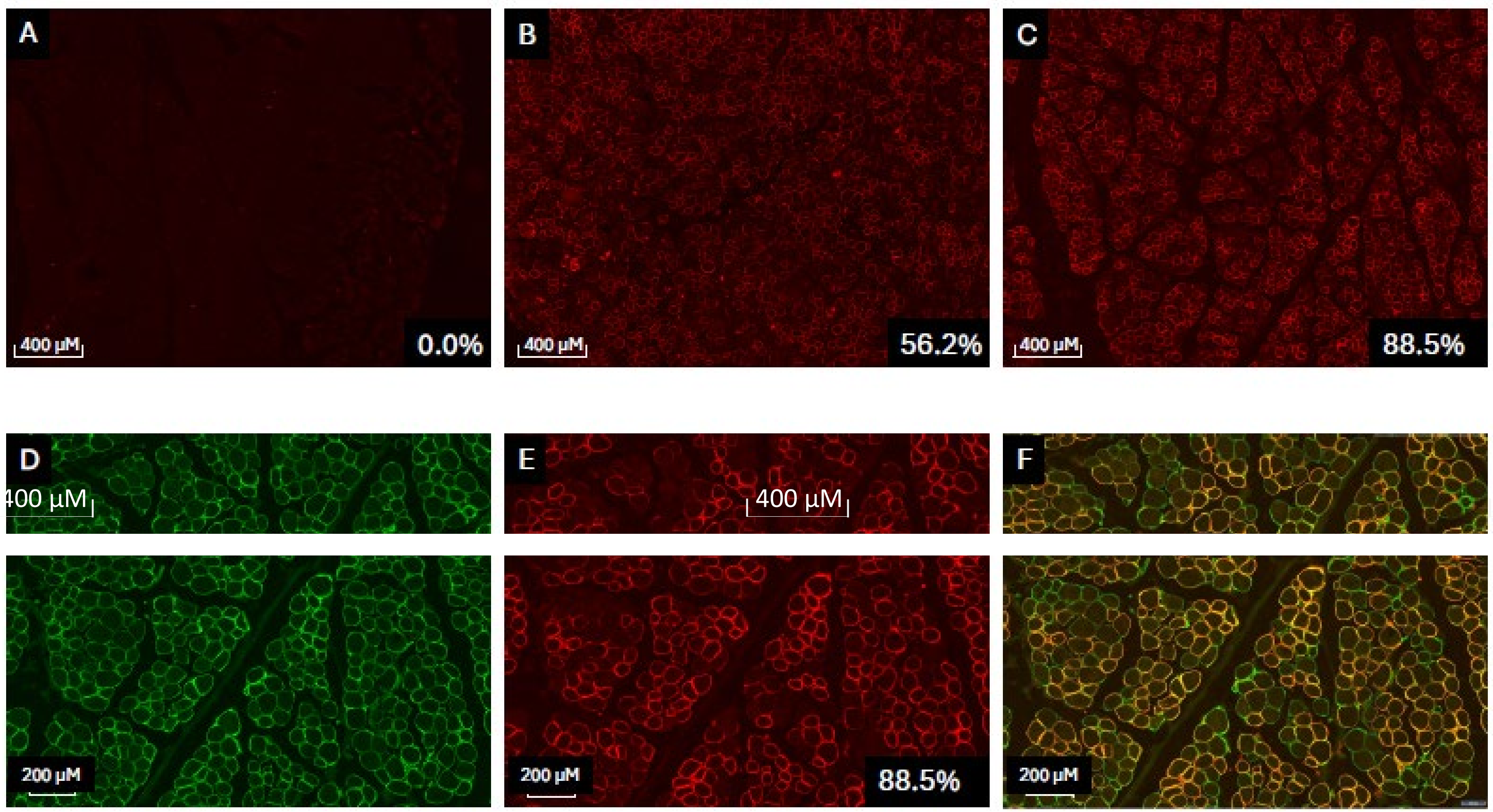


**A-C**: red (TRITC) staining against a mini-dystrophin specific antibody in a representative participant's biceps brachii tissue. This ambulatory participant was 9 years old at baseline and received the high dose of fordadistrogene movaparvovec. Tissue slices were taken from open biopsies at baseline (A), 2-months after infusion of fordadistrogene movaparvovec (B), and at 12 months after infusion (C). Percentage of fibers identified as expressing minidystrophin at the myofibril basal lamina was 0.0%, 56.2%, and 88.5%,

respectively. **D-F**: high magnification images from successive tissue slices shown in C, from 12-months post-infusion: D: green (FITC) staining identifying the myofibril basal lamina via an anti-laminin antibody; E shows red staining of mini-dystrophin; and F shows a merge of D & E.

FITC, fluorescein-5-isothiocyanate; TRITC, tetramethylrhodamine

**Figure 3. Study design.**

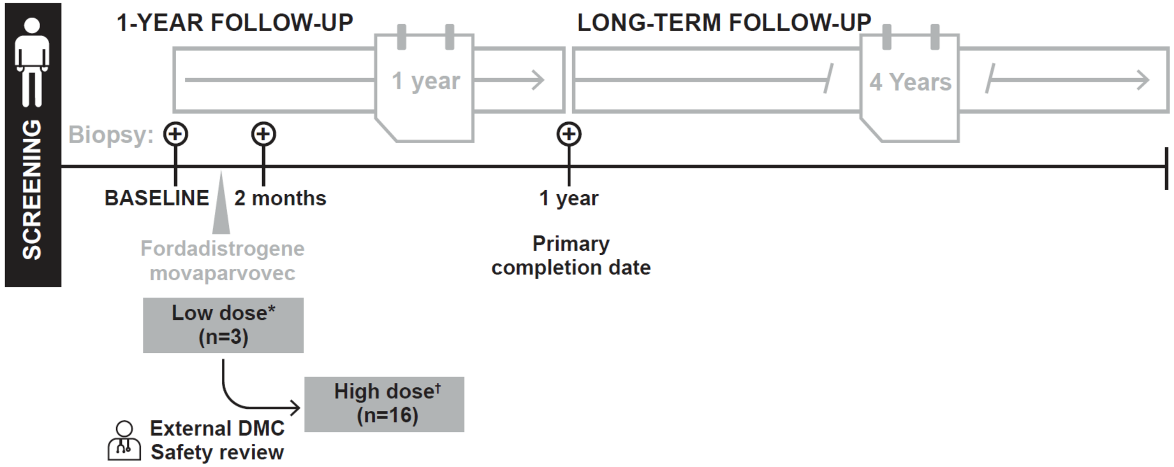


Following screening and baseline assessment (day −16), participants received a single intravenous infusion of low dose or high dose fordadistrogene movaparvovec (day 1). Enrolment and dosing within/between dose groups were initially staggered to mitigate potential risks to participants and included a formal review by an external data monitoring committee (Supplementary Methods). Over the first 1 year of follow-up (the scope of this paper), visits occurred in the clinic (weeks 2, 4, 8, 12, 26, and 52) or remotely (weeks 3, 6, 10, 15, 19, 22, 32, and 39). Muscle biopsies were collected at baseline, 2 months, and 1 year.

Blood samples were also taken at Weeks 3, 6, 10, 15, 19, 22, 32 and 39. Vital signs: 30 minutes, 1, 2, 4, 8, and 24 hours after the start of the infusion, and on Days 4, 7, 10, 14 and 30. Triplicate ECGs: approximately one hour prior to infusion of study drug, as well as upon completion of the infusion (+30 minutes) and as clinically indicated.

*Low dose was 1E14 vg/kg determined by ITR assay.

†High dose was 2E14 vg/kg determined by transgene-based assay, which is approximately equivalent to 3E14 vg/kg by ITR assay.

DMC, Data Monitoring Committee; ECG, electrocardiogram; ITR, inverted terminal repeat.
