# Supplementary material for: AAV mini-dystrophin gene therapy for Duchenne muscular dystrophy: a phase 1b trial
Source: Nat Med. 2025 Jun 27;31(8):2712–21. doi: 10.1038/s41591-025-03750-3 (PMC12353823; doi:10.1038/s41591-025-03750-3)
Supplement: Supplementary file 2 — Reporting Summary [file 41591_2025_3750_MOESM2_ESM.pdf]

nature portfolio

Corresponding author(s): Perry B. Shieh

Last updated by author(s): Jan 9, 2025

Reporting Summary

Nature Portfolio wishes to improve the reproducibility of the work that we publish. This form provides structure for consistency and transparency in reporting. For further information on Nature Portfolio policies, see our Editorial Policies and the Editorial Policy Checklist.

Statistics

For all statistical analyses, confirm that the following items are present in the figure legend, table legend, main text, or Methods section.

n/a	Confirmed	
☐	☒	The exact sample size (*n*) for each experimental group/condition, given as a discrete number and unit of measurement
☐	☒	A statement on whether measurements were taken from distinct samples or whether the same sample was measured repeatedly
☐	☒	The statistical test(s) used AND whether they are one- or two-sided<br>*Only common tests should be described solely by name; describe more complex techniques in the Methods section.*
☐	☒	A description of all covariates tested
☐	☒	A description of any assumptions or corrections, such as tests of normality and adjustment for multiple comparisons
☐	☒	A full description of the statistical parameters including central tendency (e.g. means) or other basic estimates (e.g. regression coefficient) AND variation (e.g. standard deviation) or associated estimates of uncertainty (e.g. confidence intervals)
☐	☒	For null hypothesis testing, the test statistic (e.g. F, t, r) with confidence intervals, effect sizes, degrees of freedom and P value noted<br>*Give P values as exact values whenever suitable.*
☒	☐	For Bayesian analysis, information on the choice of priors and Markov chain Monte Carlo settings
☐	☒	For hierarchical and complex designs, identification of the appropriate level for tests and full reporting of outcomes
☒	☐	Estimates of effect sizes (e.g. Cohen's *d*, Pearson's *r*), indicating how they were calculated

Our web collection on statistics for biologists contains articles on many of the points above.

Software and code

Policy information about availability of computer code

Data collection	No software was used for data collection.
Data analysis	SAS version 9.4 (SAS Institute Inc, Cary, NC, USA) and/or R version 4.1.3 (The R Foundation, Vienna, Austria).

For manuscripts utilizing custom algorithms or software that are central to the research but not yet described in published literature, software must be made available to editors and reviewers. We strongly encourage code deposition in a community repository (e.g. GitHub). See the Nature Portfolio guidelines for submitting code & software for further information.

Data

Policy information about availability of data

All manuscripts must include a data availability statement. This statement should provide the following information, where applicable:

- Accession codes, unique identifiers, or web links for publicly available datasets
- A description of any restrictions on data availability
- For clinical datasets or third party data, please ensure that the statement adheres to our policy

Upon reasonable request and subject to review, Pfizer will provide the data that support the findings of this study. Subject to certain criteria, conditions and exceptions, Pfizer may also provide access to the related individual deidentified participant data from Pfizer-sponsored global interventional clinical studies conducted for medicines, vaccines and medical devices (1) for indications that have been approved in the United States and/or European Union or (2) in programs that have been terminated (that is, development for all indications has been discontinued). Pfizer will also consider requests for the protocol, data dictionary and

statistical analysis plan. See https://www.pfizer.com/science/clinical-trials/trial-data-and-results for more information.
Data may be requested from Pfizer trials 24 months after study completion. The deidentified participant data will be made available to researchers whose proposals meet the research criteria and other conditions, and for which an exception does not apply, via a secure portal. To gain access, data requestors must enter into a data access agreement with Pfizer.

Human research participants

Policy information about studies involving human research participants and Sex and Gender in Research.

Reporting on sex and gender	All participants in this study were males.
Population characteristics	There was a total of 28 screenings completed with 24 unique ambulatory participants (four ambulatory participants were rescreened). Five unique participants did not meet eligibility criteria, with one being excluded due to the presence of neutralizing antibodies to AAV9 (other reasons for screen failure are given in Fig. 3A). A total of 19 ambulatory participants were assigned to and received study treatment (low dose, n = 3; high dose, n = 16). All ambulatory participants completed ≥1 year of follow-up. Mean±standard deviation [SD] age at dosing was 8.6±1.6 years. Mutations for ambulatory participants are summarized in Table 1, and all individual mutations are listed in Supplementary Table 1. Additional demographics and clinical characteristics for study participants are shown in Table 1 and Table 3. All participants who were enrolled and received treatment had negative results for neutralizing antibodies against AAV9 at baseline, 17 of whom were also tested and had negative results for total binding antibodies against AAV9 at baseline.<br>There were 7 non-ambulatory participants screened (4 participants did not meet eligibility criteria) and 3 non-ambulatory participants all received the high dose of fordadistrogene movaparvovec, Fig. 3B). Mean±SD age at dosing was 15.1±1.0 years. Additional demographics and clinical characteristics for non-ambulatory study participants are shown in Table 2. All participants who were enrolled and received treatment had negative results for neutralizing antibodies against AAV9 at baseline.
Recruitment	Depending on availability of investigational product, up to 22 subjects, including up to 19 ambulatory subjects and 3 non-ambulatory subjects, including those participating in the sirolimus cohort, are planned to participate in up to 5 centers in the US. Those withdrawn for reasons other than safety may be replaced at the discretion of the investigator. An internal independent team was created to validate the external control cohort in a non-biased manner to ensure interpretability of results and validity of conclusions. To minimize selection bias, study NCT03362502 eligibility criteria including age, ambulatory status, glucocorticoids use, an ability to rise from floor within 7 seconds, and cardiac function (data permitting), along with a requirement for non-missing NSAA scores at baseline and 12 months, were applied to create a subset of participants for the external control cohort.
Ethics oversight	This ongoing, non-randomized, open-label, ascending dose, phase 1b study (NCT03362502) was initiated in January 2018 at three sites in the United States (Supplementary Figure 1). It was conducted in compliance with ethical principles of the Declaration of Helsinki and all International Conference on Harmonisation Good Clinical Practice Guidelines. The protocol was approved by the relevant institutional review board/independent ethics committee at each study site (University of Utah Institutional Review Board, Duke University Health System and UCLA Medical Institutional Review Board). All participants (or parent/legal guardian) provided written informed consent.

Note that full information on the approval of the study protocol must also be provided in the manuscript.

Field-specific reporting

Please select the one below that is the best fit for your research. If you are not sure, read the appropriate sections before making your selection.

☒ Life sciences ☐ Behavioural & social sciences ☐ Ecological, evolutionary & environmental sciences

For a reference copy of the document with all sections, see nature.com/documents/nr-reporting-summary-flat.pdf

Life sciences study design

All studies must disclose on these points even when the disclosure is negative.

Sample size	The sample size of this ongoing, non-randomized, open-label, ascending dose, phase 1b study was based on clinical (rather than statistical) considerations to provide adequate safety, tolerability, and pharmacodynamic data. No statistical method was used to predetermine sample size.
Data exclusions	All data collected are included in the per-specified analysis. Full inclusion criteria are provided in the manuscript.
Replication	Independent validation was conducted to replicate the results to include in the manuscript.
Randomization	The experiments were not randomized.

Blinding	The Investigators were not blinded to allocation during experiments and outcome assessment.

Reporting for specific materials, systems and methods

We require information from authors about some types of materials, experimental systems and methods used in many studies. Here, indicate whether each material, system or method listed is relevant to your study. If you are not sure if a list item applies to your research, read the appropriate section before selecting a response.

Materials & experimental systems

n/a	Involved in the study
☐	☒ Antibodies
☒	☐ Eukaryotic cell lines
☒	☐ Palaeontology and archaeology
☒	☐ Animals and other organisms
☐	☒ Clinical data
☒	☐ Dual use research of concern

Methods

n/a	Involved in the study
☒	☐ ChIP-seq
☒	☐ Flow cytometry
☒	☐ MRI-based neuroimaging

Antibodies

Antibodies used	Antibody Description (Host/antigen/isotype/clone): Mouse anti-Mini-DYS, Mouse IgG2b, Clone A.01 25H5.H6, Source: Pfizer, Catalog # ----, Working Concentration: 10 ug/ml<br>Antibody Description (Host/antigen/isotype/clone): Anti-Laminin 2 alpha, Rat IgG1, clone: 4H8-2, Source: Abcam, Catalog # ab11576; Working Concentration: 5.5 ug/ml
Validation	• Mouse anti-Mini-DYS, Mouse IgG2b, Clone A.01 25H5.H6: This antibody was custom generated in house by Pfizer. The validation was performed using cell pellet arrays that had a range of expression level of mini-dys, from none to high levels of mini-dys expression. In addition, individual tissue samples were stained using this antibody, and a range of expected expression level (absence of staining, light and strong staining) as well as subcellular localization of mini-dys were observed in at least 80% of total individual tissue samples.<br>• Anti-Laminin 2 alpha, Rat IgG1, clone: 4H8-2: This is a commercial antibody from Abcam that is used in Laminin 2 alpha IHC. This clone 4H8-2 is the most widely used clone for Laminin 2 alpha on the market and is cited in >270 publications. Product datasheet from the manufacturer: Antibodies, Proteins, Kits and Reagents for Life Science | Abcam.

Clinical data

Policy information about clinical studies

All manuscripts should comply with the ICMJE guidelines for publication of clinical research and a completed CONSORT checklist must be included with all submissions.

Clinical trial registration	NCT03362502
Study protocol	Request Data from Vivli - Vivli
Data collection	Data collection occurred at each study site (study start date January 23, 2018; primary completion date March 28, 2022, NCT NCT03362502 ClinicalTrials.gov). Participants from 3 sites in the United States were enrolled in the trial.
Outcomes	The primary endpoints were dose-limiting adverse events, safety and tolerability through 1 year post treatment based on the incidence, severity, and causal relationship of TEAEs. These were coded using Medical Dictionary for Regulatory Activities version 24.0, with severity and relationship to treatment determined by site investigators. Other safety assessments included the incidence and magnitude of abnormal findings from clinical laboratory tests, physical and neurologic examinations, electrocardiograms, LVEF (assessed via MRI or echocardiogram), and the C-SSRS were conducted throughout the study through Week 52 (Supplementary Figure 1). The secondary endpoint was expression of mini-dystrophin in biceps brachii muscle biopsy samples. Total dystrophin concentration LC-MS and the proportion of mini-dystrophin-positive fibers (assessed by automated image analysis of immunofluorescence) were determined at baseline, 2 months, and 1 year post treatment (Supplementary Methods). The LC-MS assay used for the assessment recognize both full-length dystrophin and mini-dystrophin, while the immunofluorescent assay used a mini-dystrophin specific antibody and thereby, detects the mini-dystrophin transgene only. The term 'dystrophin' will be used to describe both forms of the protein.<br>Pre-specified exploratory functional endpoints included change from baseline to 1 year in the NSAA total score,34 time to rise from floor, time to climb four stairs (4SC), 6-minute walk distance (6MWD), time to walk/run 10 meters (10M w/r), performance of the upper limb [PUL] 2.0 and percent predicted forced vital capacity (%pFVC). Refinement of two exploratory endpoints were made: number of NSAA items (ie, skills) gained (among participants with at least one baseline individual item score of zero) and the number of skills maintained or improved (among all participants).
